# Expression Signature of lncRNAs and mRNAs in Sevoflurane-Induced Mouse Brain Injury: Implication of Involvement of Wide Molecular Networks and Pathways

**DOI:** 10.3390/ijms22031389

**Published:** 2021-01-30

**Authors:** Congshan Jiang, Thiago Arzua, Yasheng Yan, Xiaowen Bai

**Affiliations:** 1 Department of Anesthesiology, Medical College of Wisconsin, Milwaukee, WI 53226, USA; jiangcongshan@xjtu.edu.cn; 2 Department of Cell Biology, Neurobiology & Anatomy, Medical College of Wisconsin, Milwaukee, WI 53226, USA; tarzua@mcw.edu (T.A.); yashengyan@mcw.edu (Y.Y.); 3 Department of Physiology, Medical College of Wisconsin, Milwaukee, WI 53226, USA

**Keywords:** developmental neurotoxicity, sevoflurane, lncRNAs, bioinformatics analysis, molecular networks

## Abstract

Sevoflurane, one of the most commonly used pediatric anesthetics, was found to cause developmental neurotoxicity. To understand specific risk groups and develop countermeasures, a better understanding of its mechanisms is needed. We hypothesize that, as in many other brain degeneration pathways, long non-coding RNAs (lncRNAs) are involved in the sevoflurane-induced neurotoxicity. Postnatal day 7 (PD7) mice were exposed to 3% sevoflurane for 6 h. To quantify neurotoxicity in these mice, we (1) detected neural apoptosis through analysis of caspase 3 expression level and activity and (2) assessed long-term learning ability via the Morris water maze at PD60. To elucidate specific mechanisms, profiles of 27,427 lncRNAs and 18,855 messenger RNAs (mRNAs) in mouse hippocampi were analyzed using microarray assays. Sevoflurane-induced abnormal lncRNA and mRNA expression-associated function pathways were predicted by bioinformatic analysis. We found that sevoflurane induced significant neurotoxicity, causing acute neuroapoptosis and abnormal expression of 148 mRNAs and 301 lncRNAs on PD7 in mouse hippocampus. Additionally, exposed mice exhibited impaired memory on PD60. Bioinformatic analysis predicted that the dysregulated mRNAs, which are highly correlated with their co-expressed dysregulated lncRNAs, might be involved in 34 neurodegenerative signaling pathways (e.g., brain cell apoptosis and intellectual developmental disorder). Our study reveals for the first time that neonatal exposure to 3% sevoflurane induces abnormal lncRNA and mRNA expression profiles. These dysregulated lncRNAs/mRNAs form wide molecular networks that might contribute to various functional neurological disease pathways in the hippocampus, resulting in the observed acute apoptosis and impaired long-term memory.

## 1. Introduction

During the rapid synaptogenesis period [1], the brain goes through an intensive developmental process, rendering it relatively vulnerable to environmental stressors such as anesthetics [2]. Emerging evidence from animal and clinical studies suggests that anesthetic can induce developmental neurotoxicity (AIDN), raising serious concerns on the use of the anesthetics in pediatric populations [3,4,5,6,7,8]. Acute AIDN was evidenced by neuroapoptosis (programmed neural death) [9], widespread neurodegeneration [10], reduced neurogenesis [11] and synaptogenesis [12]. The long-lasting outcomes manifest as cognitive dysfunction and abnormal behavior [5,10,13].

So far, volatile sevoflurane has been considered as one of the most commonly used pediatric anesthetics in clinical practice due to its favorable physicochemical properties [13]. However, evidence from a C57BL/6 mouse study showed that exposure to the clinically relevant dose of 3% sevoflurane for 6 h could lead to neuroapoptosis immediately after anesthesia, and impaired social memory and interaction [14]. Similar adverse effects conferred by sevoflurane were observed in multiple animal studies [15,16,17]. Furthermore, a retrospective study found significant associations between exposure of anesthetics such as sevoflurane and poorer intelligence quotients in children with hypoplastic left heart syndrome [18]. The underlying molecular mechanism is of great interest, yet not understood.

Long non-coding RNAs (lncRNAs) with the lengths longer than 200 nucleotides [19] are emerging to play a great part in various neurodegenerative diseases [20]. Several recent studies using various doses and lengths of sevoflurane exposure suggested the importance of lncRNAs in sevoflurane-induced developmental neurotoxicity. For instance, one recent study from Zhang et al. showed that sevoflurane exposure for 3 continuous days (2 h per day) to postnatal day (PD) 6 mice (with a dose of 3%) could lead to the reduced expression of lncRNA Rik-203. The similarly decreased Rik-203 expression was also observed in the sevoflurane-treated mouse embryonic stem cells (ESCs)-derived neuron differentiation culture. Moreover, the downregulation of lncRNA Rik-203 expression could impair neural differentiation of ESCs [21].

These studies provide insights into the importance of lncRNAs in AIDN, indicating that further investigation into this area is valuable. According to widely accepted understanding, the minimum alveolar concentration (MAC) of sevoflurane is 3.3 ± 0.2% in human neonates and 3.2 ± 0.1% in human infants under 6 months [22,23]. Moreover, as we mentioned before, previous evidence demonstrated that the exposure to 3% sevoflurane for 6 h in mice could lead to neuroapoptosis immediately after anesthesia, and impaired social memory and interaction [14]. Hence, in our current study, we used an unbiased microarray to analyze the lncRNA and mRNA expression profiling in mouse hippocampal tissues following 3% sevoflurane (6 h) treatment as well as to predict lncRNA/mRNAs-involved signaling networks in sevoflurane-induced developmental neurotoxicity by informatics analysis.

## 2. Results

### 2.1. Sevoflurane Induces Neuroapoptosis in the Neonatal Mouse Hippocampi in a Time-Dependent Manner

**Figure 1:**
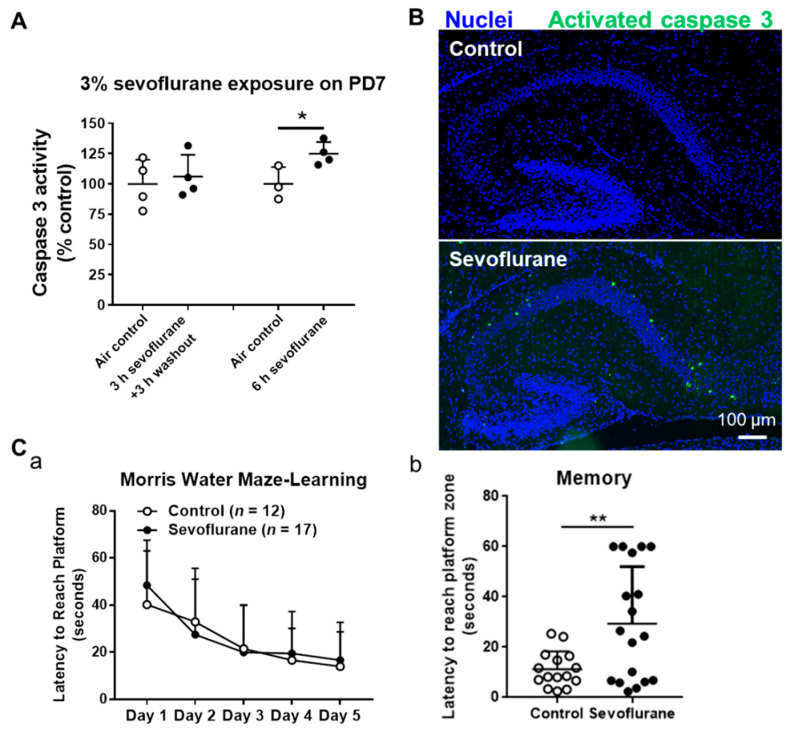
Neonatal 3% sevoflurane exposure to postnatal day 7 (PD7) mice for 6 h induces acute apoptosis in hippocampi and results in impaired memory of PD60 adult mice. (**A**) Caspase 3 activity assay shows an increase in neuroapoptosis in the PD7 mouse hippocampi immediately after exposure to 3% sevoflurane for 6 h. Data were presented with mean ± SD (*n* = 4 per group; *: *p* < 0.05. (**B**) Fluorescence images of the immunofluorescence-stained brain sagittal sections demonstrate that sevoflurane treatment increased activated caspase 3-positive cells (green) in the PD7 mouse hippocampi. Scale bar: 100 μm. Blue represents Hoechst 33342-stained cell nuclei. (**C**) Morris water maze showed that neonatal sevoflurane exposure impaired memory capacity but not learning ability in PD60 mice (*n* = 12 for control and *n* = 17 for sevoflurane group); **: *p* < 0.01.

PD7 mice were exposed to 3% sevoflurane for 3 and 6 h. We did not observe increased caspase 3 activity (an apoptotic marker) in the mouse hippocampi 3 h after completing 3 h sevoflurane exposure. However, the caspase 3 activity was increased in hippocampal tissues of 6 h sevoflurane-treated mice (*p* = 0.034) after 6 h of sevoflurane exposure (Figure 1A). The immunofluorescence staining also demonstrated that there was obvious activated caspase 3 staining in the cornus ammonis 1 (CA1) and CA2 subfields of hippocampi after exposure to 3% sevoflurane for 6 h (26 in sevoflurane vs. one apoptotic cell/hippocampal section in sevoflurane vs. control group) (Figure 1B). These data suggest that neonatal sevoflurane exposure time-dependently induces apoptosis in mouse hippocampal tissues. 

### 2.2. Exposure of Sevoflurane to PD7 Mice Leads to Impaired Memory Capacity in PD60 Adult Mice

We analyzed the learning and memory abilities of P60 mice, both controls and mice exposed to 3% sevoflurane for 6 h at PD7, using the Morris water maze. The data showed that the latency for PD60 to reach the platform during the entire training period from day 1 to day 5 in the sevoflurane exposure group was not significantly different from the control group, indicating that sevoflurane does not affect the long-term learning ability of mice (Figure 1C). However, the neonatal sevoflurane-treated PD60 took a longer time (*p* = 0.004) to reach the platform zone on day 6 of the memory test than control mice, indicating that developing sevoflurane exposures result in impaired memory capacity in adult mice. 

### 2.3. Sevoflurane Exposure on PD7 Induces the Alteration of mRNA Profiles in Mouse Hippocampi

**Figure 2:**
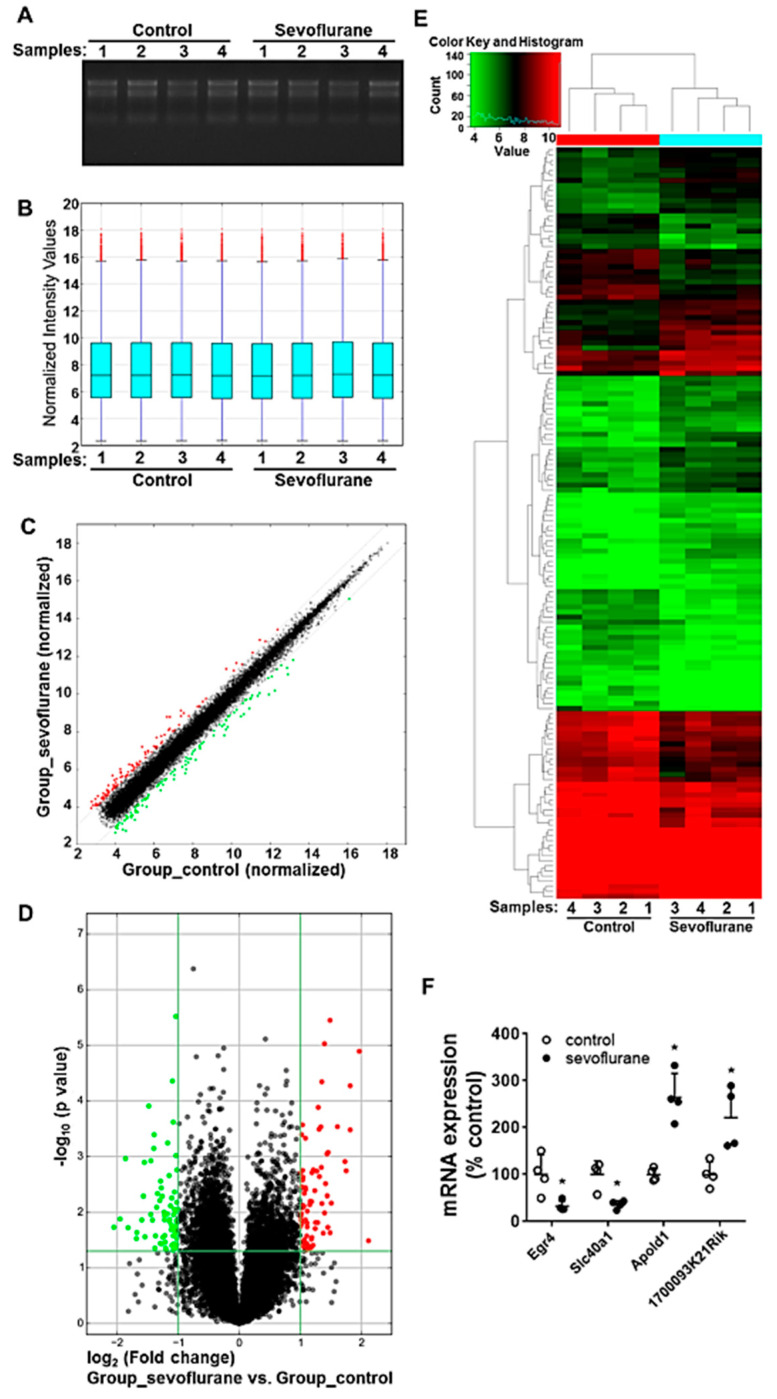
Exposure to 3% sevoflurane for 6 h induces the differentially expressed mRNA profiles in the mouse hippocampi (4 samples per group) on PD7. (**A**) The denaturing agarose gel electrophoresis image for quality control of total RNAs isolated from 8 mouse hippocampi. (**B**) The box plots show the consistent distributions of normalized mRNA signal intensity values. For each box, the central line represents the median, whereas the tails represent the upper and lower quartiles. (**C**) The scatter plot displays the consistency of normalized mRNA intensity values from control and sevoflurane groups. (**D**) The volcano plot illustrates the differentially expressed mRNAs between control and sevoflurane groups. (**C**,**D**) Red dots: differentially up-regulated mRNAs, green dots: differentially down-regulated mRNAs, *p* < 0.05, fold change above 2, compared with control. (**E**) Heatmap and hierarchical clustering displaying the expression profiles of sevoflurane-induced differentially expressed mRNAs in mouse hippocampi (*p* < 0.05). Each row represents the relative expression of each gene. Image with higher resolution was shown in Supplementary Figure S1. (**F**) RT-qPCR validation of the expression of 4 randomly selected dysregulated mRNAs (Egr4, Slc40a1, Apold1, and 1700093K21Rik) from microarray assay. Data presented with mean ± SD; *: *p* < 0.05.

To evaluate the alteration of mRNA expression following the sevoflurane exposure with a high-throughput and hypothesis-free strategy, the mRNA expression profiling was analyzed using the microarray assay. The denaturing agarose gel electrophoresis image shows that total RNAs used for microarray assay exhibit good quality, as evidenced by the integrity without degradation and no genomic DNA contamination (Figure 2A). The distribution of normalized microarray intensity values of each sample was displayed in a box plot showing the spread and centers of a dataset. The results indicate that the similar distribution of the data from different groups and the microarray dataset were suitable for further analysis (Figure 2B). The scatter plot indicates that the normalized expression data are highly consistent between control and sevoflurane groups (person correlation: 0.9932), except for minor part of sevoflurane-induced dysregulated mRNAs (Figure 2C). Volcano plot and heatmap demonstrated that there were 77 up-regulated and 71 down-regulated mRNAs (fold change beyond ± 2, *p* < 0.05) among all 18,855 detected mRNAs following sevoflurane exposure (Figure 2D,E, Supplementary Figure S1). Four randomly selected dysregulated mRNAs according to the microarray dataset were further validated using reverse transcription-quantitative polymerase chain reaction (RT-qPCR) (Figure 2F). The data from RT-qPCR (all *p* = 0.021) are consistent with the microarray dataset. All microarray data are included in the GEO database (GSE155770). This dysregulated gene list is included in Supplementary Table S1. Notably, thirty-nine (e.g., Dgcr14, Egr4, Ptprr et al.) of these 148 sevoflurane-dysregulated mRNAs have been previously suggested to be involved in nervous system activities in health and disorders. For instance, sevoflurane exposure reduced SLPI and microtubule associated serine/threonine kinase 2 (MAST2). SLPI is necessary and sufficient for axon regeneration in the central nervous system [24]. MAST2 could inhibit the neurite outgrowth and lysophosphatidic acid-induced neurite retraction [25].

### 2.4. Sevoflurane Exposure-Induced Dysregulated mRNA Are Potentially Involved in Neurotoxicity-Related Signalling

**Figure 3:**
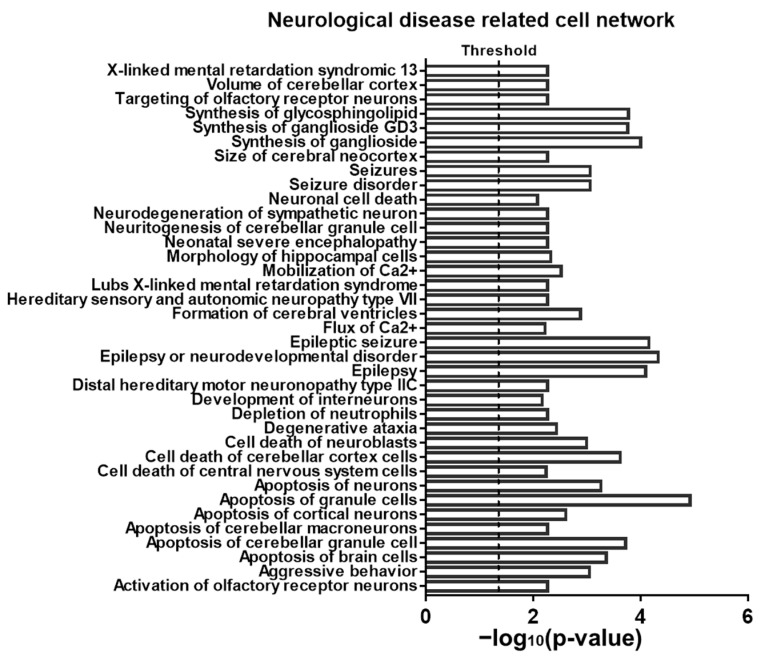
Bioinformatics analysis of neurological disease-related cell networks for sevoflurane-induced dysregulated mRNAs using ingenuity pathway analysis (IPA) software. This figure lists the 37 neurological cell networks (listed alphabetically) from the total number of 500 influenced by the sevoflurane-induced dysregulated mRNA profiles. Such disease mechanism-related signaling networks were predicted according to the uploaded result of sevoflurane-induced altered mRNA list from the microarray dataset based on the existing archives of IPA database. The threshold value of 1.3 represents significantly (*p* < 0.05) implicated cell networks.

The brain’s development is a complex process. Sevoflurane not only induced neuroapotosis, as shown in Figure 1, but also possible disturbed other brain development events. To find the potential molecular signaling pathways that are involved in apoptosis as well as in other anesthetic-induced pathology changes, we used ingenuity pathway analysis (IPA) software to analyze the pathways and potential implication of these sevoflurane-induced differentially expressed mRNAs (*p* < 0.05 and fold change beyond ± 2) in diseases and functions. According to the results, among all the involved diseases and functions, cell death and survival, neurological disease, as well as organismal injury and abnormalities are the top three most significantly implicated categories. More specifically, in the subsets there are 30 neurological diseases and functions (further detailed in Supplementary Table S2). To better understand how these dysregulated mRNAs might participate in these mentioned neurological diseases and functions, we used IPA to analyze the related cell networks and found that 37 signaling networks are neurologically related (Figure 3).

### 2.5. Sevoflurane Induces the Alteration of lncRNA Expression in PD7 Mouse Hippocampi

**Figure 4:**
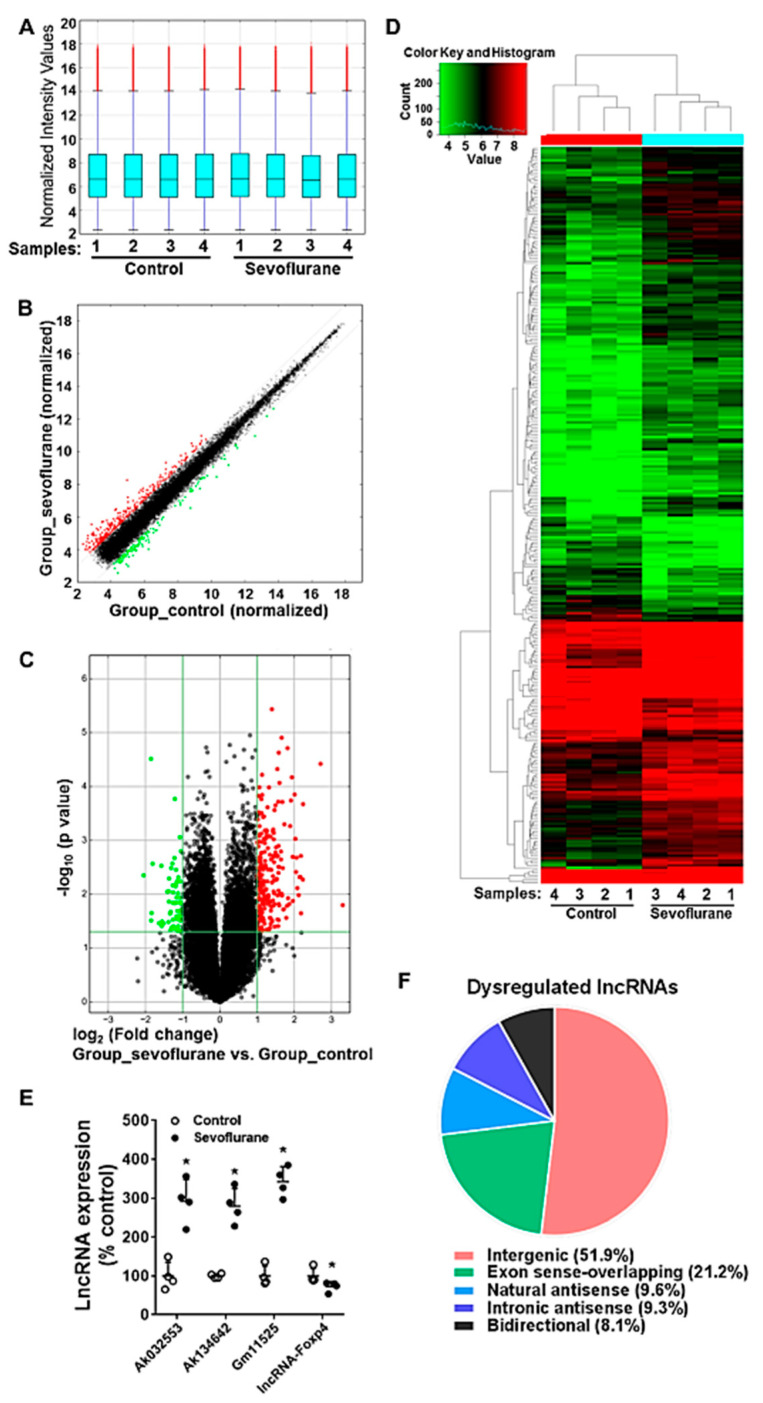
Exposure to 3% sevoflurane for 6 h induces the differentially expressed long non-coding RNAs (lncRNAs) profiles in the PD7 mouse hippocampi (4 samples per group). (**A**) The box plots showing the similar distribution of normalized lncRNA signal intensity values from 8 mouse hippocampi. For each box, the central line represents the median of lncRNA intensity values, whereas the tails represent the upper and lower quartiles. (**B**) The scatter plot displaying the mostly consistence of normalized lncRNA intensity values from control and sevoflurane groups. (**C**) The volcano plot illustrating the differentially expressed lncRNAs between air control and sevoflurane groups. (**B**,**C**) Red dots: differentially up-regulated lncRNAs; green dots: differentially down-regulated lncRNAs, *p* < 0.05, fold change ≥ 2, compared against control. (**D**) Heatmap and hierarchical clustering displaying the expression profiles of sevoflurane-induced differentially expressed lncRNAs in mouse hippocampi (*p* < 0.05). Each row represents the relative expression of one lncRNA gene. Image with higher resolution was shown in Supplementary Figure S2. (**E**) RT-qPCR validation of eight randomly selected dysregulated lncRNAs from array data (including Ak032553, Ak134642, Gm11525, and lncRNA Foxp4), bar: mean ± SD, *: *p* < 0.05. (**F**) Pie chart shows various types of sevoflurane-induced dysregulated lncRNAs.

The lncRNA expression profiling was also detected by using the microarray assay. The box plot shows that the distribution of normalized intensity values of all samples from control and sevoflurane groups is similar, and thus the dataset was suitable for further analysis (Figure 4A). The scatter plot indicates that the normalized expression data from control and sevoflurane groups are highly consistent with each other, except for a minor part of dysregulated lncRNAs (person correlation: 0.9908) (Figure 4B). The volcano plot and heatmap demonstrate that there were 234 up-regulated and 67 down-regulated lncRNAs significantly altered during sevoflurane exposure (fold change beyond ±2 and *p* < 0.05) among all 27,427 detected lncRNAs (Figure 4C,D, Supplementary Figure S2). Four randomly selected dysregulated lncRNAs according to the microarray dataset were further validated by using RT-qPCR (Figure 4E). The results show that the RT-qPCR detected expression profile (all *p* = 0.021) was consistent with the findings from the microarray dataset for these dysregulated lncRNAs. According to the intersection with its nearby protein-coding genes, lncRNAs can be classified into several types, such as intergenic, exon sense-overlapping, natural antisense, intronic antisense, and bidirectional lncRNAs [26]. The results show that intergenic, exon sense-overlapping, natural antisense, intronic antisense, and bidirectional lncRNAs represent 51.9%, 21.1%, 9.6%, 9.3%, and 8.1% of all 301 dysregulated lncRNAs, respectively (Figure 4F). The data suggested that such various types of lncRNAs are likely to perform their functions via diverse mechanisms.

### 2.6. Sevoflurane Exposure Results in the Dysregulated lncRNAs and Their Corresponding co-Expressed mRNAs in Mouse Hippocampi

To explore the potential signaling pathway of sevoflurane-induced dysregulated lncRNAs, we further dissected these dysregulated lncRNAs for subgroups based on their genomic location and action mechanisms in accordance with the conjugated protein coding genes. As we found out, a total of 9.6% sevoflurane-dysregulated lncRNAs are natural antisenses. It was revealed that natural antisense transcripts were able to negatively regulate the conjugated sense transcript [27], thus, such natural antisense lncRNAs could play an important role in the regulation of their conjugated protein coding genes if they are both dysregulated after sevoflurane exposure. The data indicate that there is no dysregulated natural antisense type of lncRNA and mRNA pair after sevoflurane exposure. In addition, lncRNAs can induce transcription-dependent activation or repression of its nearby protein-coding genes [28]. We found that six lncRNAs together with their nearby coding genes (distance within 300 kb) were dysregulated (*p* < 0.05, fold change beyond ± 2) following sevoflurane exposure, including Gm16287-*Ubr4*, Ak048274-*Igfbp5*, Ak081961-*Igfbp5*, Ak137249-*Mal1*, Ak138713-*Xlr5b,* and A730032A03Rik-*Slpi* (described in Table 1).

### 2.7. The Predicted Signaling Networks Formed between Sevoflurane Exposure-Induced Altered lncRNAs and mRNAs

**Figure 5:**
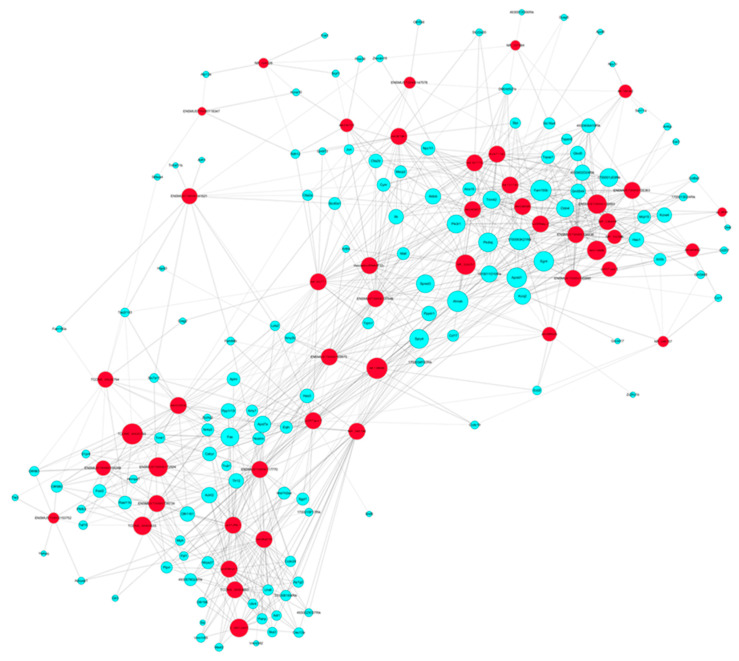
Coding (mRNA) and non-coding gene (lncRNA) co-expression (CNC) networks image based on the bioinformatics analysis of the top 50 ranked sevoflurane-induced dysregulated lncRNAs and their associated mRNAs. Blue node: coding genes (mRNAs). Red node: non-coding genes (lncRNAs). Solid line: positive Pearson correlation coefficient (PCC). Dotted line: negative PCC. Note: An amplified figure with a higher resolution is shown in Supplementary Figure S3.

With the knowledge of these dysregulated lncRNAs following sevoflurane exposure, we went through the entire list of 301 lncRNAs and tried to find out any possible clues from the previous literature. However, previous reports for these 301 dysregulated lncRNAs are very scarce, and functions of only six lncRNAs of them were studied. Specifically, the actions of these six lncRNAs in neurodevelopment, nervous system, and neurobiology are not known. With the help of bioinformatics, we can predict the possible signaling of dysregulated lncRNAs based on the pathway implication of their highly correlated co-expressed mRNAs. Such bioinformatics analysis was achieved with the coding (mRNAs) and non-coding (lncRNAs) gene co-expression (CNC) and IPA analysis. CNC analysis explored the potential regulation interaction relationship of lncRNAs and their high correlated co-expressed mRNAs (absolute value of Pearson correlation coefficients, PCC above 0.9, *p*-value less than 0.05, and false-discovery rate FDR less than 1). The CNC image further demonstrated that there were 47 sevoflurane-dysregulated lncRNAs highly correlated with 132 sevoflurane-dysregulated mRNAs, generating 985 lncRNA–mRNA interaction pairs in hippocampi from the neonatal mice the CNC networks plotted in Figure 5 (Supplemental Figure S4 is the same as Figure 5 with higher magnification), and each sevoflurane-dysregulated lncRNA–mRNA interaction pair was documented in Supplementary Table S4. 

**Figure 6:**
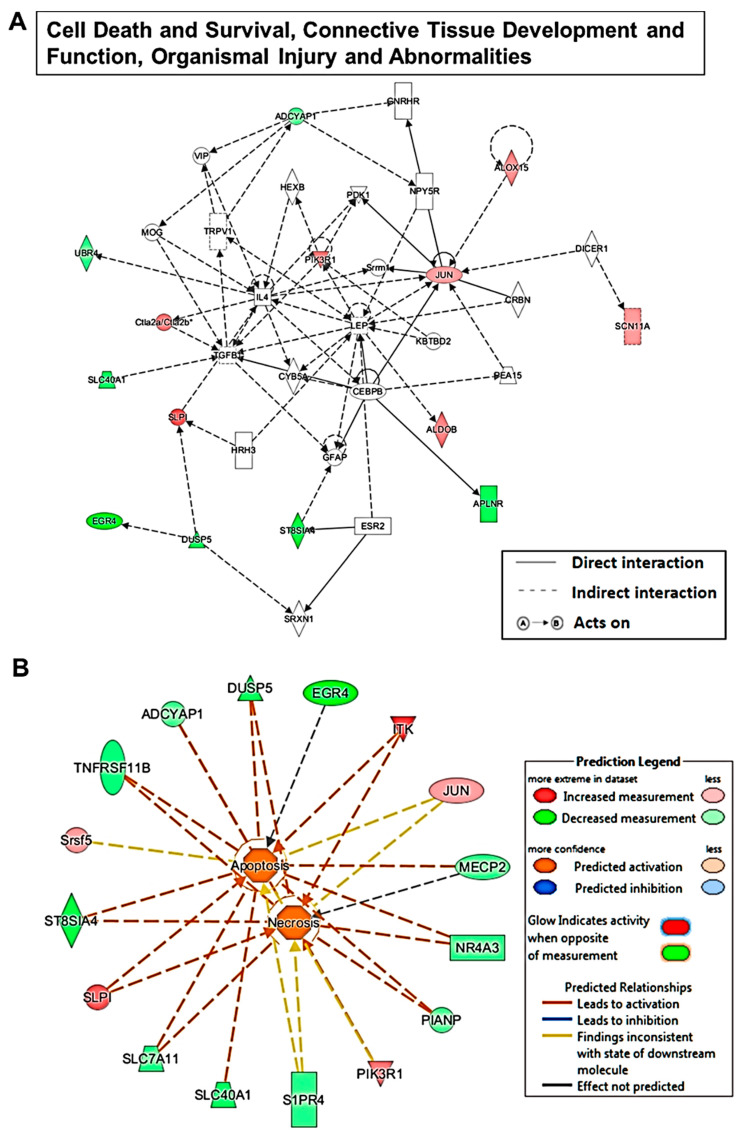
Bioinformatics analysis of sevoflurane altered lncRNA-related pathways based on the dysregulated lncRNA co-expressed dysregulated mRNAs using ingenuity pathway analysis (IPA) software. The figure displays how each of the sevoflurane-dysregulated mRNAs, which are highly correlated with their corresponding co-expressed dysregulated lncRNAs, are involved in top disease and function (**A**), as well as apoptosis and necrosis networks (**B**). Each symbol represents one individual gene. Solid and dotted lines show a direct and indirect connection between genes. Green symbols indicate downregulation and red indicate upregulation following sevoflurane exposure. The abbreviations of the genes were defined in Supplemental Table S3.

These highly correlated mRNAs could be an important candidate target gene and hence participate in the lncRNA-mediated mechanism during sevoflurane-induced developmental neurotoxicity. The lncRNA pathways were predicted by analyzing the sevoflurane-dysregulated mRNAs which were highly correlated with their co-expressed dysregulated lncRNAs using the IPA algorithm. The results show that among all the diseases and functions, the dysregulated lncRNAs are predicted to be most likely involved in cell death and survival, connective tissue development and function, organismal injury and abnormalities. Within such pathways, there were 14 lncRNA co-expressed related genes (mRNAs) dysregulated following sevoflurane exposure. These 14 mRNAs’ regulative network signaling is depicted in Figure 6A. We also included representative lncRNA/mRNA networks, showing how each of the dysregulated mRNAs and their corresponding co-expressed dysregulated lncRNAs involved. For example, these regulative networks are predicted to be involved in apoptosis and necrosis with outstanding activation scores. Figure 6 and Supplementary Table S5 described how these molecules get involved in both pathways, and especially how they are likely to talk to each other. Moreover, among all the involved diseases and functions, the dysregulated lncRNAs were predicted to be implicated in 34 neurological diseases and functions, possibly by regulating their corresponding co-expressed dysregulated mRNAs (Table 2).

## 3. Discussion

Sevoflurane, one of the most commonly used pediatric anesthetics, was found to cause developmental neurotoxicity. To understand specific risk groups and develop countermeasures of sevoflurane-induced developmental neurotoxicity, a better understanding of its mechanisms is needed. Our study reveals for the first time that neonatal exposure to 3% sevoflurane induces abnormal lncRNA and mRNA expression profiles. These dysregulated lncRNAs/mRNAs form wide molecular networks that might contribute to various functional neurological diseases pathways in the hippocampus, resulting in the observed acute apoptosis and impaired long-term memory. 

mRNAs and long noncoding RNAs (lncRNAs) are two important RNAs participating in transcription regulation. Our study showed that there were 148 dysregulated mRNAs following sevoflurane exposure (Figure 2). Thirty-nine of them have been previously suggested to be involved in nervous system activities in health and disorders. These unbiased assays suggest that many genes and molecular networks might be involved in sevoflurane-induced developmental neurotoxicity. In particular, three (MeCP2, Ctla2a, and Jun) of these dysregulated mRNAs have already been indicated as being associated with sevoflurane-induced neurotoxicity in previous studies [29,30,31,32].

Unlike mRNAs, lncRNAs are not translated into proteins. Many lncRNAs have been implicated as tuners of cell fate. There is little information about the actions of lncRNAs in sevoflurane neurotoxicity. Our results reveal that there were 301 lncRNAs altered in neonatal mouse hippocampal tissue following sevoflurane exposure. All sevoflurane-induced dysregulated lncRNAs have not been studied for their association with brain development and function. Thus, we dissected signaling pathways of these lncRNAs’ involvement in the neurotoxicity by bioinformatic analysis of sevoflurane-induced dysregulated genes (mRNAs) that were highly co-expressed with these lncRNAs. We found that these dysregulated lncRNAs are closely associated with 34 neurological diseases and functions (Table 2) and most significantly implicated in some general cell networks including apoptosis and necrosis. These dysregulated genes are predicted to form wide regulative signaling networks that are involved in the neurotoxicity (Figure 4 and Figure 5, and Supplementary Table S5). Convincing pre-clinical data support the perception that sevoflurane exposure could lead to excessive neuroapoptosis, poor behavior, and cognitive function in developing mice [14,33]. This type of excessive neural cell death brings fewer functional neural units to support the fundamental well-organized network for long-term cognition [34]. Our study supports this perception, and our data provide a novel understanding that sevoflurane exposure might cause the above-mentioned neurotoxicity by affecting the wide molecular networks and pathways including those related to intellectual developmental disorder by regulating the lncRNAs and mRNAs involved.

When compared with protein-coding genes (mRNAs), lncRNAs play distinct roles in various locations such as nuclear, nucleolar, cytoplasmic, and even individual cellular compartments. The lncRNA-involved gene regulation mechanisms are complicated through various actions such as regulation of transcription, genome organization, and architecture of the nucleus [35]. The dysregulated lncRNAs and their neighboring dysregulated protein-coding mRNAs were widely considered significantly important for molecular mechanism during various disorders. Such dysregulated lncRNA–mRNA pairs were very much likely to serve as the core component of regulating axis of signaling. We found that six lncRNAs together with their nearby coding genes were dysregulated following sevoflurane exposure (Table 1). Interestingly, the dysregulated lncRNA–mRNA pair (A730032A03Rik-*Slpi)* was also found in the intravenous anesthetic propofol-treated mouse hippocampus [36]. Several studies showed that Slpi was strongly up-regulated in response to CNS injury and that exogenous administration of Slpi is neuroprotective [37]. It is likely that lncRNA A730032A03Rik performs an important function in the sevoflurane-induced neurotoxicity, and manipulation of A730032A03Rik/Slpi signaling might be one of the alternative strategies for combatting neurotoxicity. 

Through comparison of lncRNAs from our study and others using different sevoflurane doses, exposure frequency, and models, we found different lncRNA expression profiles. For instance, so far, there are two reported sevoflurane exposure-related lncRNA profiling studies. One in vitro study found that 2.4% sevoflurane (6 h) could lead to abnormal lncRNA expressions (1183 up-regulated and 1416 down-regulated) in hippocampal neural stem cells from neonatal SD rats [38]. Another microarray study showed that 2.3% sevoflurane (6 h) resulted in 31 dysregulated lncRNAs and 25 mRNAs in PD7 mouse hippocampi [39]. These 31 lncRNAs do not match any of our 301 lncRNAs dysregulated in the PD7 mouse hippocampi following 3% sevoflurane exposure for 6 h. Additionally, Gm15621 was down-regulated during sevoflurane exposure (1, 2, and 4%) for 6 h in primary hippocampal neuron cells from neonatal mice [40]. However, we did not observe the sevoflurane-induced changes of the Gm15621 expression. These sevoflurane-induced lncRNA profiles from different studies suggest the importance of lncRNAs in the neurotoxicity. Such differences in lncRNA profiling might have resulted from the diverse sevoflurane exposure conditions and experiment models used in these studies. 

So far, preclinical studies and cell culture models have demonstrated that most general anesthetics such as ketamine, propofol, and sevoflurane induce developmental neurotoxicity with common acute pathological changes in brains such as apoptosis and long-term cognition dysfunction [7,37,41,42]. Different anesthetics might share similar lncRNA mechanisms in AIDN. Previously, our group identified multiple dysregulated lncRNAs and mRNAs in hippocampi from mice exposed to propofol [36]. There were twenty mRNAs (including *Slpi, 1700001J03Rik, Alox15, Trim42, Ctla2b, Ly6c1, 1810011O10Rik, Hao1, Itk, 1700093K21Rik, Ctla2a, Sis, Rdh12, Mall, Adh1, Egr4, Dusp5, Olfr961, Has3* and Spred3) and twenty-four lncRNAs (such as Gm11525, AK045106, AK132723 et al.) that were observed dysregulated in both propofol- [36] and sevoflurane-treated (Supplementary Table S3) PD7 mouse brains. Such similarity in these lncRNA and mRNA expression profiles suggests the common roles in anesthetic-induced neurotoxicity. These genes might be promising candidates for AIDN study in future. 

One of the caveats of this study lies in the relevance of our animal model to clinical setting. We used 7-day old mouse models and exposure duration is six hours. Thus, the sevoflurane exposure length for mice in our study may not be equal to the same duration in human clinical practice. A second limitation of our study is that the functional roles of the candidate genes of interest in the progression of the anesthetic neurotoxicity are needed for further investigation. Finally, our current study evaluated the acute apoptosis and long-term cognition dysfunction as the end point. Since brain development is complicated and involved in many developmental events. It is possible that sevoflurane exposure causes detrimental effects on neurogenesis and cell migration as well as synaptogenesis. In addition to decreased memory capacity, the potential of sevoflurane-induced subtle behavioral changes cannot be excluded.

Collectively, this study has a high degree of clinical relevance. Many young children are exposed to sevoflurane for surgical purposes. The observed neuroapoptosis and decreased memory capacity raise concerns on the safe use of anesthetics in children and pregnant women. The unbiased microarray analyses and extensive bioinformatic analysis suggest the involvement of wide molecular networks and pathways in the sevoflurane-induced developmental neurotoxicity. Our findings provide valuable insights into lncRNA mechanisms underlying sevoflurane-induced developmental neurotoxicity, and illuminate the new research on the promising lncRNA and mRNA candidates for functional study in AIDN as well as the potential targets for neuroprotection. 

## 4. Materials and Methods

### 4.1. Animal Studies

C57BL/6 mice (Jackson Laboratories, Bar Harbor, ME, USA) were housed in the specific pathogen-free animal facility at the MCW. PD7 mice were exposed to sevoflurane when the developing brain is the most vulnerable for AIDN. Both genders of mice were used and randomly distributed into sevoflurane or control groups.

### 4.2. Anesthetic Exposure

PD7 mice were exposed to either sevoflurane (3%) in a chamber with 40% O_2_ or 40% O_2_ alone as an air control for 3 or 6 h. During anesthesia, all pups were placed on a heating pad and rectal temperature was maintained at 37 ± 1 °C. For the “3-h exposure +3-h washout” group, mice were kept on the heating pad for another 3 h after the 3 h of anesthesia. After that, the mice were immediately euthanized for hippocampus harvest. For the 6 h exposure studies, the mice were divided for different experiments. Some mice were sacrificed for tissue harvest immediately after the exposure, while others were placed back to home cages used for Morris water maze assay of cognitive function on PD60. 

MouseOx Plus Pulse Oximeter (Starr Life Sciences Corp, Oakmont, PA, USA) were used to measure oxygen saturation. As shown in Supplementary Figure S4, sevoflurane did not influence the oxygen saturation. The depth of anesthesia was monitored during the sevoflurane exposure by checking the righting reflex and pinprick response of mice. The mice were in deep anesthesia status one hour after sevoflurane exposure, as evidenced by no voluntary movement and no response to righting reflex and pinprick. It is vital to the results that the blood and tissue samples from the control group were free from anesthetics exposure. Hence, PD7 mouse pups were decapitated quickly with sharp scissors without anesthesia, and hippocampal tissues from 4 animals per group were collected for RNA and protein assays.

### 4.3. Caspase 3 Acitivity Assay

Caspase 3 activity was detected in mouse hippocampal tissues by using a caspase 3 colorimetric assay kit (Sigma-Aldrich, St. Louis, MO, USA) following the manufacturer’s instructions, as we previously described [36].

### 4.4. Immunofluorescence Staining

Perfusion was performed in 4 animals from both groups, and the whole brain tissues were collected for histology examination of apoptosis as we previously described [42]. Briefly, mice were subjected to perfusion-fixation using 10% zinc formalin fixation solution (Richard-Allan Scientific, San Diego, CA, USA) through the left ventricle once the animal reached a surgical plane of anesthesia with isoflurane. Paraffin-embedded brain tissue blocks were then cut into 4 µm-thick sagittal sections. Deparaffinized sections were then subjected to antigen retrieval by incubation in target retrieval solution (Dako, Santa Clara, CA, USA) for 20 min in a boiling steamer. Then, sections were subjected to immunofluorescent staining using a primary antibody rabbit anti-activated caspase 3 (Asp175, apoptosis marker; Cell Signaling 9664) and secondary antibody Alexa Fluor 488-conjugated donkey anti-rabbit IgG (H + L) (Thermo Fisher Scientific, Waltham, MA, USA, A-21206). The slides were stained for nuclei with Hoechst 33,342 (Thermo Fisher Scientific, Waltham, MA, USA). The slides were imaged using an Olympus Fluorescent Slide Scanner and OlyVIA2.4 software (Olympus Corporation of the Americas, Center Valley, PA, USA).

### 4.5. Cognitive Function Assay

The Morris water maze was used to determine the potential cognitive (learning and memory) dysfunction in PD60 mice with single sevoflurane exposure on PD7. Both memory capacity and learning ability of mice were evaluated in 12 control and 17 sevoflurane-exposed mice. Briefly, a circular polypropylene pool (100 cm in diameter and 20 cm in height) was filled with water plus a non-toxic white paint, rendering it opaque. On the pool rim, four points were designated (north, east, south, and west), dividing the pool into four quadrants (NE, NW, SW, SE). The water was changed and its temperature was checked daily to be 20–22 °C. A platform (8 × 8 cm) was positioned at the center of the SE quadrant, with the standing area submerged ~2 cm below the surface of the water. Each mouse was tracked via EthoVision XT (Noldus Information Technology, Washington, DC, USA) starting from a random start point until it reached the platform, or after 60 s. If unable to find the platform in 60 s, the mouse was guided by the investigator. Trials were repeated 4 times per day, with an interval of 5 min, for 5 days. On the 6th day, the platform was removed from the pool in order to test the animal’s memory. The mouse was positioned in the water from a new starting point and was allowed to swim for 60 s while being tracked. Latency to escape was defined as the time that the animal took to find the platform, or the platform zone on the 6th day; other measures, such as total swim distance, speed, time spent in each quadrant were also measured. At the end of the experiments, all animals were euthanized by carbon dioxide inhalation.

### 4.6. RNA Extraction

Mouse hippocampi were lysed in Qiazol reagent (Qiagen company, Hilden, Germany) and the total RNA was extracted by using a phenol-chloroform method as we previously described [43]. The possible contamination of genomic DNA was further eliminated by using DNA-free^TM^ kit (Ambion, Life technologies, Carlsbad, CA, USA). The quantity and quality of the total RNA was detected by using Nanodrop 3000 (Thermoscientific, Waltham, MA, USA). The total RNA was used for microarray and RT-qPCR assays to determine lncRNA and mRNA expression.

### 4.7. Microarray Assay

Five micrograms of hippocampal RNA was used for mouse LncRNA Microarray (v3.0) assay. Both the microarray assay and data analysis for lncRNA signaling networks were performed by Arraystar Inc. (Rockville, MD, USA). This microarray assay covers 27,427 lncRNAs and 18,855 mRNAs in the hippocampus from each mouse. In the microarray assay, a series of quality control assays were included. Nanodrop ND-100 spectrophotometer and standard denaturing agarose gel electrophoresis were used to validate the RNA quality and quantity, while the box plot and scatter plot were used to assess the signal reproducibility. The RNA was synthesized to cDNA, and hybridized to the microarray probes for fluorescence intensity scanning. Detection bias between signal intensities associated with different mRNA sequences is cancelled out for the same mRNA in the comparison between the groups/conditions. The significantly dysregulated lncRNAs and mRNAs were filtered by using the volcano plot (expressing fold change beyond ± 2.0 and *p* < 0.05 between control and sevoflurane groups). The heat map and hierarchical clustering displays all the target values for the differentially expressed lncRNAs and mRNAs, the samples were clustered together and the color key on the top left stands for the log2-transformed fragments per kilo base of transcripts per million mapped reads value. The entire dataset are deposited in GEO database (GSE155770). The results from microarray assays were further validated by using RT-qPCR assays.

### 4.8. RT-qPCR

Four dysregulated lncRNAs (including Ak032553, Ak134642, Gm11525, lncRNA Foxp4) and mRNAs (including Egr4, Slc40a1, Apold1, 1700093K21Rik) were randomly selected, and further validated by using RT-qPCR to verify the authenticity of microarray assay data as previously described [43]. Briefly, cDNA was synthesized by using RevertAid™ First Strand cDNA Synthesis Kit (Thermoscientific, Waltham, MA, USA) from a total RNA of 2.5 μg (with mixed primer of oligo d(T) and random hexamer). In the qPCR assays, the cDNA, PowerUp™ SYBR™ Green Master Mix (Appliedbiosystems, Foster City, CA, USA), primers (listed in Supplementary Table S6), and pure water (Qiagen, Valencia, CA, USA) were mixed for reaction. PCR triplicates were used, and qPCR reactions were performed by using the QuantStudio™ 6 Real-Time PCR machine (Appliedbiosystems, Foster City, CA, USA). The specificity of the PCR reaction was checked with the melting curves of PCR product at the end of reaction. The mean cycle threshold (Ct) values from the PCR triplicates were used, and the raw data for mRNA expression were further normalized against endogenous control Actb and finally analyzed by using 2^−ΔΔCt^ calculation.

### 4.9. IPA of Dysregulated Mrnas and Related Pathways

The IPA (Qiagen, Valencia, CA, USA) is a potent bioinformatics tool for predicting the disease mechanisms and canonical physiological signaling pathways according to the uploaded differentially expressed gene lists (mRNAs with fold change above ± 2.0 and *p* < 0.05 between groups) [44]. Networks and pathway analysis was achieved according to the present understanding of individual gene’s participation in established pathways based on the literature on the IPA database. Based on our microarray dataset for dysregulated mRNAs, we obtained a collection of prediction of possible implication in various neurological diseases and functions (with Fisher’s exact test *p* < 0.05 calculated in IPA database). In addition, according to our present outcome of sevoflurane-induced neurotoxicity, we further narrowed down our scale for the specific cell networks that are related to neurological disorders. 

### 4.10. Networks Analysis of Dysregulated lncRNAs-Related Pathways and lncRNA-mRNA Interactions

lncRNA pathways were analyzed based on the 50 top-ranked dysregulated lncRNAs and all dysregulated mRNAs by using bioinformatics algorithm as previously described [41]. The top 50 lncRNAs were selected according to the criteria of *p* < 0.01 and the top-ranking absolute fold change value (sevoflurane group compared against control). The lncRNA and mRNA regulation interaction was displayed by CNC analysis. PCC between the top 50 ranked dysregulated lncRNA and all dysregulated mRNA expression were calculated. Co-expressed dysregulated lncRNA-mRNAs pairs with absolute values of PCC above 0.9, *p* values less than 0.05 and FDR less than 1 were filtered and used for the generation of the CNC image, plotted by using Cytoscape V2.8.3 software. The lncRNA pathway was predicted by analyzing the sevoflurane-dysregulated mRNAs which are highly correlated with their co-expressed dysregulated lncRNAs using IPA algorithm.

### 4.11. Statistics

The statistical differences between two groups were calculated by using SPSS software (version 24, IBM SPSS Statistics, Armonk, NY, USA), and figures were plotted by using GraphPad Prism (version 7.0, San Diego, CA, USA). An unpaired independent t test was used to analyze the dysregulated lncRNAs and mRNAs in the microarray assay, while for the RT-qPCR results and caspase 3 activity assay, the Mann–Whitney U test was performed. Sample size was decided based on the pilot data from our lab and the previous similar studies [37]; hence, *n* = 4 was chosen for gene analysis and apoptosis, while *n* = 10 or *n* = 15 was chosen for the behavior test. Data were presented as mean ± standard deviation (SD) of mice, and two-tailed *p* < 0.05 was considered as statistically significant.

## Figures and Tables

**Table 1:** Dysregulated lncRNAs and their nearby coding mRNAs in PD7 mouse brains following 3% sevoflurane exposure for 6 h.

lncRNA Symbol	lncRNA Strand	lncRNA				mRNA Symbol	mRNA Strand	Nearby Coding mRNA			
		*p*-Value ^1^	Fold Change	Regulation	FDR			*p*-Value	Fold Change	Regulation	FDR
Gm16287	−	0.00024	2.4	up	0.099	Ubr4	+	0.042	2.1	down	0.32
Ak048274	+	0.014	2.4	up	0.22	Igfbp5	-	0.011	2.3	down	0.24
Ak081961	+	0.000038	6.5	up	0.057	Igfbp5	-	0.011	2.3	down	0.24
Ak137249	−	0.0019	2.9	up	0.14	Mall	-	0.00013	2.5	up	0.078
Ak138713	−	0.00093	2.3	up	0.12	Xlr5b	+	0.027	2.2	up	0.28
A730032A03Rik	+	0.039	2.9	down	0.28	Slpi	-	0.0018	3.4	up	0.17

Note: ^1^ *p* values and expression fold changes are displayed against air control group. FDR: false-discovery rate. The full gene names of the sevoflurane-induced dysregulated mRNAs are detailed in Supplementary Table S3.

**Table 2:** The sevoflurane exposure (6 h)-induced dysregulated lncRNAs associated with neurological diseases and functions.

Diseases or Functions Annotation	*p*-Value	mRNAs	Highly Correlated Co-Expressed lncRNAs
Apoptosis of granule cells	0.00000606	ADCYAP1, FAS, FRAT1, JUN, MECP2	ENSMUST00000153752, uc012fts.1, AK084576, TCONS_00020793, ENSMUST00000169975, AK143771, TCONS_00020794, ENSMUST00000137546, ENSMUST00000135739, ENSMUST00000172524, uc007guo.1, uc012fts.1, ENSMUST00000155268, uc009mzl.1, mouselincRNA0733-, TCONS_00000533, ENSMUST00000117770, AK084576, AK032934, NR_024326, AK143771, AK167175, AK081961, AK030101, ENSMUST00000137546, mouselincRNA0733- NR_024257, AK131720, AV471140, AK143771, AK167175, AK081961, AK030101, ENSMUST00000137546, mouselincRNA0733-
Epileptic seizure	0.000033	ADCYAP1, CCR1, DUSP5, EGR4, HAS3, HSPB3, JUN, NR4A3	ENSMUST00000153752, uc012fts.1,AK084576, ENSMUST00000145890, AK045892, NR_046157, AK131720, AK167175, ENSMUST00000134436, NR_024257, NR_036459, AK131720, uc009alp.2, ENSMUST00000126693, AK045957, AK086925, AV471140, ENSMUST00000155363, AK036595, AK014666, AK167175, ENSMUST00000145890, ENSMUST00000137546, AK045892, uc007uqa.1, NR_045190, NR_024257, ENSMUST00000169975, AK143771, ENSMUST00000137546, ENSMUST00000172524, uc007guo.1, uc012fts.1, mouselincRNA0733-, ENSMUST00000117770, AK084576, NR_045190, ENSMUST00000169975, AK143771, AK167175, AK081961, AK030101, ENSMUST00000137546, mouselincRNA0733-, ENSMUST00000169975, AK143771, TCONS_00020794, ENSMUST00000137546, ENSMUST00000135739, ENSMUST00000172524, uc012fts.1, uc009mzl.1, TCONS_00000533
Epilepsy or neurodevelopmental disorder	0.0000338	ADCYAP1, AHNAK, BRPF1, CCR1, DUSP5, EGR4, GLI3, HAS3, HSPB3, JUN, MECP2, NR4A3, SCN11A	ENSMUST00000153752, uc012fts.1, AK084576, ENSMUST00000134436, NR_024257, NR_036459, AK131720, uc009alp.2, ENSMUST00000169975, AK045957, AK086925, AK143771, AK036595, AK167175, ENSMUST00000145890, ENSMUST00000137546, AK138866, NR_102396, uc007guo.1, mouselincRNA0733-, AK084576, NR_045190, AK081961, AK030101, NR_024326, ENSMUST00000145890, AK045892, NR_046157, AK131720, AK167175, ENSMUST00000134436, NR_024257, NR_036459, AK131720, uc009alp.2, ENSMUST00000126693, AK045957, AK086925, AV471140, ENSMUST00000155363, AK036595, AK014666, AK167175, ENSMUST00000145890, ENSMUST00000137546, AK045892, uc007uqa.1, NR_045190, ENSMUST00000135739, uc007guo.1, NR_024257, ENSMUST00000169975, AK143771, ENSMUST00000137546, ENSMUST00000172524, uc007guo.1, uc012fts.1, mouselincRNA0733-, ENSMUST00000117770, AK084576, NR_045190, ENSMUST00000169975, AK143771, AK167175, AK081961, AK030101, ENSMUST00000137546, mouselincRNA0733-, NR_024257, AK131720, AV471140, AK143771, AK167175, AK081961, AK030101, ENSMUST00000137546, mouselincRNA0733-, ENSMUST00000169975, AK143771, TCONS_00020794, ENSMUST00000137546, ENSMUST00000135739, ENSMUST00000172524, uc012fts.1, uc009mzl.1, TCONS_00000533, AK149342
Epilepsy	0.000035	ADCYAP1, CCR1, DUSP5, EGR4, GLI3, HAS3, HSPB3, JUN, MECP2, NR4A3, SCN11A	ENSMUST00000153752, uc012fts.1, AK084576, ENSMUST00000145890, AK045892, NR_046157, AK131720, AK167175, ENSMUST00000134436, NR_024257, NR_036459, AK131720, uc009alp.2, ENSMUST00000126693, AK045957, AK086925, AV471140, ENSMUST00000155363, AK036595, AK014666, AK167175, ENSMUST00000145890, ENSMUST00000137546, AK045892, uc007uqa.1, NR_045190, ENSMUST00000135739, uc007guo.1, NR_024257, ENSMUST00000169975, AK143771, ENSMUST00000137546, ENSMUST00000172524, uc007guo.1, uc012fts.1, mouselincRNA0733-, ENSMUST00000117770, AK084576, NR_045190, ENSMUST00000169975, AK143771, AK167175, AK081961, AK030101, ENSMUST00000137546, mouselincRNA0733-, NR_024257, AK131720, AV471140, AK143771, AK167175, AK081961, AK030101, ENSMUST00000137546, mouselincRNA0733-, ENSMUST00000169975, AK143771, TCONS_00020794, ENSMUST00000137546, ENSMUST00000135739, ENSMUST00000172524, uc012fts.1, uc009mzl.1, TCONS_00000533, AK149342
Apoptosis of cerebellar granule cell	0.000134	FRAT1, JUN, MECP2	NR_024326, AK143771, AK167175, AK081961, AK030101, ENSMUST00000137546, mouselincRNA0733-,NR_024257, AK131720, AV471140, AK143771, AK167175, AK081961, AK030101, ENSMUST00000137546, mouselincRNA0733-
Cell death of cerebellar cortex cells	0.000174	FAS, FRAT1, JUN, MECP2	TCONS_00020793, ENSMUST00000169975, AK143771, TCONS_00020794, ENSMUST00000137546, ENSMUST00000135739, ENSMUST00000172524, uc007guo.1, uc012fts.1, ENSMUST00000155268, uc009mzl.1, mouselincRNA0733-, TCONS_00000533, ENSMUST00000117770, AK084576, AK032934, NR_024326, AK143771, AK167175, AK081961, AK030101, ENSMUST00000137546, mouselincRNA0733-, NR_024257, AK131720, AV471140, AK143771, AK167175, AK081961, AK030101, ENSMUST00000137546, mouselincRNA0733-
Apoptosis of brain cells	0.00022	ADCYAP1, ALOX15, FAS, FRAT1, JUN, MECP2	ENSMUST00000153752, uc012fts.1, AK084576, AK131720, uc009alp.2, AK045957, AV471140, ENSMUST00000155363, AK036595, AK167175, AK081961, ENSMUST00000137546, mouselincRNA0733-, TCONS_00020793, ENSMUST00000169975, AK143771, TCONS_00020794, ENSMUST00000137546, ENSMUST00000135739, ENSMUST00000172524, uc007guo.1, uc012fts.1, ENSMUST00000155268, uc009mzl.1, mouselincRNA0733-, TCONS_00000533, ENSMUST00000117770, AK084576, AK032934, NR_024326, AK143771, AK167175, AK081961, AK030101, ENSMUST00000137546, mouselincRNA0733-, NR_024257, AK131720, AV471140, AK143771, AK167175, AK081961, AK030101, ENSMUST00000137546, mouselincRNA0733-
Seizure disorder	0.000386	ADCYAP1, CCR1,DUSP5, EGR4, GLI3, HAS3, HSPB3, JUN, MECP2, NR4A3, SCN11A, ST8SIA4	ENSMUST00000153752, uc012fts.1, AK084576, ENSMUST00000145890, AK045892, NR_046157, AK131720, AK167175, ENSMUST00000134436, NR_024257, NR_036459, AK131720, uc009alp.2, ENSMUST00000126693, AK045957, AK086925, AV471140, ENSMUST00000155363, AK036595, AK014666, AK167175, ENSMUST00000145890, ENSMUST00000137546, AK045892, uc007uqa.1, NR_045190, ENSMUST00000135739, uc007guo.1, NR_024257, ENSMUST00000169975, AK143771, ENSMUST00000137546, ENSMUST00000172524, uc007guo.1, uc012fts.1, mouselincRNA0733-, ENSMUST00000117770, AK084576, NR_045190, ENSMUST00000169975 AK143771, AK167175, AK081961, AK030101, ENSMUST00000137546, mouselincRNA0733-, NR_024257, AK131720, AV471140, AK143771, AK167175, AK081961, AK030101, ENSMUST00000137546, mouselincRNA0733-, ENSMUST00000169975, AK143771, TCONS_00020794, ENSMUST00000137546, ENSMUST00000135739, ENSMUST00000172524, uc012fts.1, uc009mzl.1, TCONS_00000533, AK149342, ENSMUST00000118347, TCONS_00020794
Seizures	0.000487	ADCYAP1, CCR1, DUSP5, EGR4, HAS3, HSPB3, JUN, MECP2, NR4A3, SCN11A, ST8SIA4	ENSMUST00000153752, uc012fts.1, AK084576, ENSMUST00000145890, AK045892, NR_046157, AK131720, AK167175, ENSMUST00000134436, NR_024257, NR_036459, AK131720, uc009alp.2, ENSMUST00000126693, AK045957, AK086925, AV471140, ENSMUST00000155363, AK036595, AK014666, AK167175, ENSMUST00000145890, ENSMUST00000137546, AK045892, uc007uqa.1, NR_045190, NR_024257, ENSMUST00000169975, AK143771, ENSMUST00000137546, ENSMUST00000172524, uc007guo.1, uc012fts.1, mouselincRNA0733-, ENSMUST00000117770, AK084576, NR_045190, ENSMUST00000169975, AK143771, AK167175, AK081961, AK030101, ENSMUST00000137546, mouselincRNA0733-, NR_024257, AK131720, AV471140, AK143771, AK167175, AK081961, AK030101, ENSMUST00000137546, mouselincRNA0733-, ENSMUST00000169975, AK143771, TCONS_00020794, ENSMUST00000137546, ENSMUST00000135739, ENSMUST00000172524, uc012fts.1, uc009mzl.1, TCONS_00000533, AK149342, ENSMUST00000118347, TCONS_00020794
Apoptosis of cortical neurons	0.00153	ALOX15, FAS, JUN, MECP2	AK131720, uc009alp.2, AK045957, AV471140, ENSMUST00000155363, AK036595, AK167175, AK081961, ENSMUST00000137546, mouselincRNA0733-, TCONS_00020793, ENSMUST00000169975, AK143771, TCONS_00020794, ENSMUST00000137546, ENSMUST00000135739, ENSMUST00000172524, uc007guo.1, uc012fts.1, ENSMUST00000155268, uc009mzl.1, mouselincRNA0733-, TCONS_00000533, ENSMUST00000117770, AK084576, AK032934, AK143771, AK167175, AK081961, AK030101, ENSMUST00000137546, mouselincRNA0733-, NR_024257, AK131720, AV471140, AK143771, AK167175, AK081961, AK030101, ENSMUST00000137546, mouselincRNA0733-
Hereditary sensory and autonomic neuropathy type VII	0.00479	SCN11A	AK149342
Zappella variant Rett syndrome	0.00479	MECP2	NR_024257, AK131720, AV471140, AK143771, AK167175, AK081961, AK030101, ENSMUST00000137546, mouselincRNA0733-
Blepharophimosis, ptosis, and epicanthus inversus type II with Duane retraction syndrome	0.00479	FOXL2	TCONS_00020793, TCONS_00020794, ENSMUST00000135739, ENSMUST00000172524, uc007guo.1, ENSMUST00000153752, uc012fts.1, ENSMUST00000155268, TCONS_00000533, AK032934
Kallmann syndrome type 17	0.00479	SPRY4	ENSMUST00000134436, AK131720, uc009alp.2, ENSMUST00000169975, AK045957, AK086925, AK143771, AK036595, ENSMUST00000145890, ENSMUST00000137546, AK138866, uc007guo.1, uc012fts.1, mouselincRNA0733-, ENSMUST00000117770, AK084576, NR_045190
Susceptibility to X-linked autism type 3	0.00479	MECP2	NR_024257, AK131720, AV471140, AK143771, AK167175, AK081961, AK030101, ENSMUST00000137546, mouselincRNA0733-
Distal hereditary motor neuronopathy type IIC	0.00479	HSPB3	ENSMUST00000169975
Susceptibility to hypogonadotropic hypogonadism type 17	0.00479	SPRY4	ENSMUST00000134436, AK131720, uc009alp.2, ENSMUST00000169975, AK045957, AK086925, AK143771, AK036595, ENSMUST00000145890, ENSMUST00000137546, AK138866, uc007guo.1, uc012fts.1, mouselincRNA0733-, ENSMUST00000117770, AK084576, NR_045190
Neurodegeneration of sympathetic neuron	0.00479	JUN	AK143771, AK167175, AK081961, AK030101, ENSMUST00000137546, mouselincRNA0733-
Hypogonadotropic hypogonadism 17 without anosmia	0.00479	SPRY4	ENSMUST00000134436, AK131720, uc009alp.2, ENSMUST00000169975, AK045957, AK086925, AK143771, AK036595, ENSMUST00000145890, ENSMUST00000137546, AK138866, uc007guo.1, uc012fts.1, mouselincRNA0733-, ENSMUST00000117770, AK084576, NR_045190
X-linked mental retardation syndromic 13	0.00479	MECP2	NR_024257, AK131720, AV471140, AK143771, AK167175, AK081961, AK030101, ENSMUST00000137546, mouselincRNA0733-
Neonatal severe encephalopathy	0.00479	MECP2	NR_024257, AK131720, AV471140, AK143771, AK167175, AK081961, AK030101, ENSMUST00000137546, mouselincRNA0733-
Intellectual developmental disorder with dysmorphic facies and ptosis	0.00479	BRPF1	AK081961, AK030101, NR_024326
Degeneration of cholinergic fibers	0.00479	JUN	AK143771, AK167175, AK081961, AK030101, ENSMUST00000137546, mouselincRNA0733-
Autosomal recessive intellectual developmental disorder 68	0.00479	TRMT1	AK143771, TCONS_00020794, uc012fts.1, ENSMUST00000155268, TCONS_00000533, ENSMUST00000117770, AK032934
Familial episodic pain syndrome type 3	0.00479	SCN11A	AK149342
Lubs X-linked mental retardation syndrome	0.00479	MECP2	NR_024257, AK131720, AV471140, AK143771, AK167175, AK081961, AK030101, ENSMUST00000137546, mouselincRNA0733-
Hypothalamic hamartoma	0.00479	GLI3	ENSMUST00000135739, uc007guo.1
Anosmia	0.00908	SLC7A11, SPRY4	ENSMUST00000141521, ENSMUST00000135739, uc007guo.1, ENSMUST00000117770, AK032934, ENSMUST00000134436, AK131720, uc009alp.2, ENSMUST00000169975, AK045957, AK086925, AK143771, AK036595, ENSMUST00000145890, ENSMUST00000137546, AK138866, uc007guo.1, uc012fts.1, mouselincRNA0733-, ENSMUST00000117770, AK084576, NR_045190
Lack of inferior colliculus	0.00957	GLI3	ENSMUST00000135739, uc007guo.1
Apoptosis of somatotrophs	0.00957	FAS	TCONS_00020793, ENSMUST00000169975, AK143771, TCONS_00020794, ENSMUST00000137546, ENSMUST00000135739, ENSMUST00000172524, uc007guo.1, uc012fts.1, ENSMUST00000155268, uc009mzl.1, mouselincRNA0733-, TCONS_00000533, ENSMUST00000117770, AK084576, AK032934
Neurodegeneration of substantia nigra pars compacta	0.00957	SLC7A11	ENSMUST00000141521, ENSMUST00000135739, uc007guo.1, ENSMUST00000117770, AK032934
Familial Angelman syndrome	0.00957	MECP2	NR_024257, AK131720, AV471140, AK143771, AK167175, AK081961, AK030101, ENSMUST00000137546, mouselincRNA0733-
Misrouting of thalamocortical axons	0.00957	ST8SIA4	ENSMUST00000118347, TCONS_00020794
Apoptosis of lactotropes	0.00957	FAS	TCONS_00020793, ENSMUST00000169975, AK143771, TCONS_00020794, ENSMUST00000137546, ENSMUST00000135739, ENSMUST00000172524, uc007guo.1, uc012fts.1, ENSMUST00000155268, uc009mzl.1, mouselincRNA0733-, TCONS_00000533, ENSMUST00000117770, AK084576, AK032934

Note: The full gene names of the sevoflurane-induced dysregulated mRNAs and lncRNAs are detailed in Supplementary Table S3.

## Data Availability

All microarray data are deposited in GEO database (GSE155770).

## Supplementary Materials

Supplementary Materials can be found at https://www.mdpi.com/1422-0067/22/3/1389/s1. Supplementary Figure S1. This figure is the amplified Figure 2E (with higher resolution). Supplementary Figure S2. This figure is the amplified Figure 3D (with higher resolution). Supplementary Figure S3. This figure is the amplified Figure 4 (with higher resolution). Supplementary Figure S4. Sevoflurane exposure does not alter oxygen saturation. Oxygen saturation was measured with MouseOx Plus Pulse Oximeter after 1 to 6 h of 3% sevoflurane exposure in mice. *n* = 5–8. Supplementary Table S1. Literature summary of sevoflurane-induced dysregulated mRNAs that are previously reported to be relevant to neurodevelopment in health and disorders. Supplementary Table S2. The bioinformatics analysis of predicted neurological diseases and functions of the sevoflurane-induced dysregulated mRNA profiles analyzed using ingenuity pathway analysis software. Supplementary Table S3. The full gene name of sevoflurane-induced dysregulated mRNAs and lncRNAs. Supplementary Table S4. Sevoflurane-dysregulated highly correlated lncRNA-mRNA interaction pairs (absolute Pearson correlation coefficient above 0.9). Supplementary Table S5. The bioinformatics analysis of co-expressed sevoflurane-dysregulated lncRNA and mRNAs involved in apoptosis and necrosis signaling networks. Supplementary Table S6. Sequence information for primers.

## Abbreviations

**Table:**

AIDN	anesthetics-induced developmental neurotoxicity
CA	cornus ammonis
CNC	coding and non-coding gene co-expression
Ct	cycle threshold
CNS	central nervous system
ESCs	embryonic stem cells
FDR	false-discovery rate
GABA	γ-aminobutyric acid
IPA	ingenuity pathway analysis
lncRNAs	long non-coding RNAs
mRNAs	messenger RNAs
LTD	long-term depression
NSC	neural stem cells
PCC	Pearson correlation coefficient
PD	postnatal day
RT-qPCR	reverse transcription-quantitative polymerase chain reaction
SD	standard deviation
SD (rat)	Sprague-Dawley (rat)
WNT5A-AS	wingless-type mouse mammary tumor virus integration site family member 5a-antisense

Note: The full gene names of the sevoflurane-induced dysregulated mRNAs and lncRNAs are listed in Supplementary Table S6.
