# Supplementary material for: Expression Signature of lncRNAs and mRNAs in Sevoflurane-Induced Mouse Brain Injury: Implication of Involvement of Wide Molecular Networks and Pathways
Source: Int J Mol Sci. 2021 Jan 30;22(3):1389. doi: 10.3390/ijms22031389 (PMC7869012; doi:10.3390/ijms22031389)
Supplement: Supplementary file 1 [file ijms-22-01389-s001.zip › ijms-1088385-supplementary/2. Supplementary Materials.docx]

**Supplementary Figures**

**
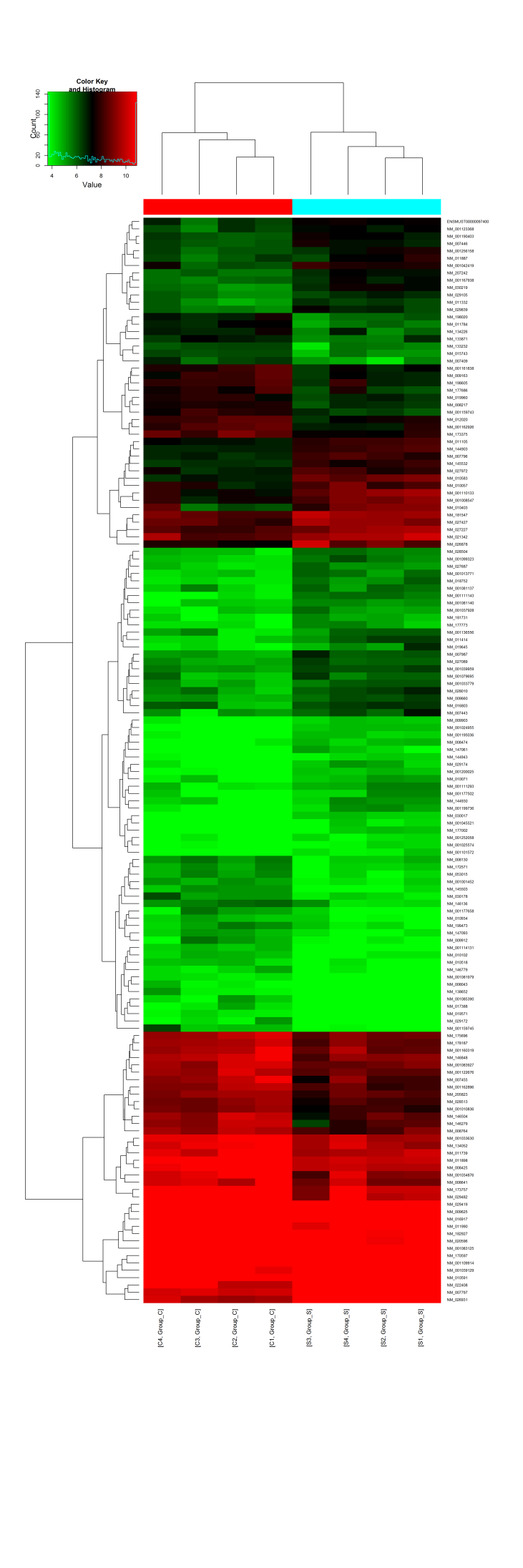
**

**Supplementary Figure S1**. This Figure is the amplified Figure 2E (with higher resolution).

**
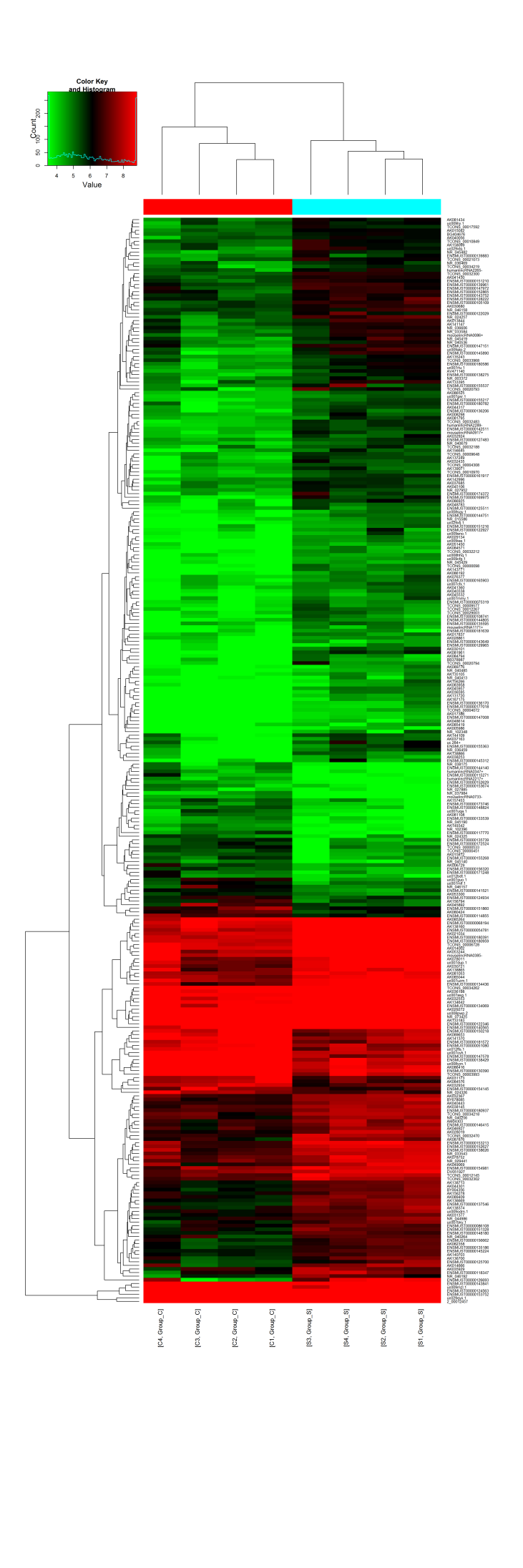
**

**Supplementary Figure S2**. This Figure is the amplified Figure 3D (with higher resolution).

**
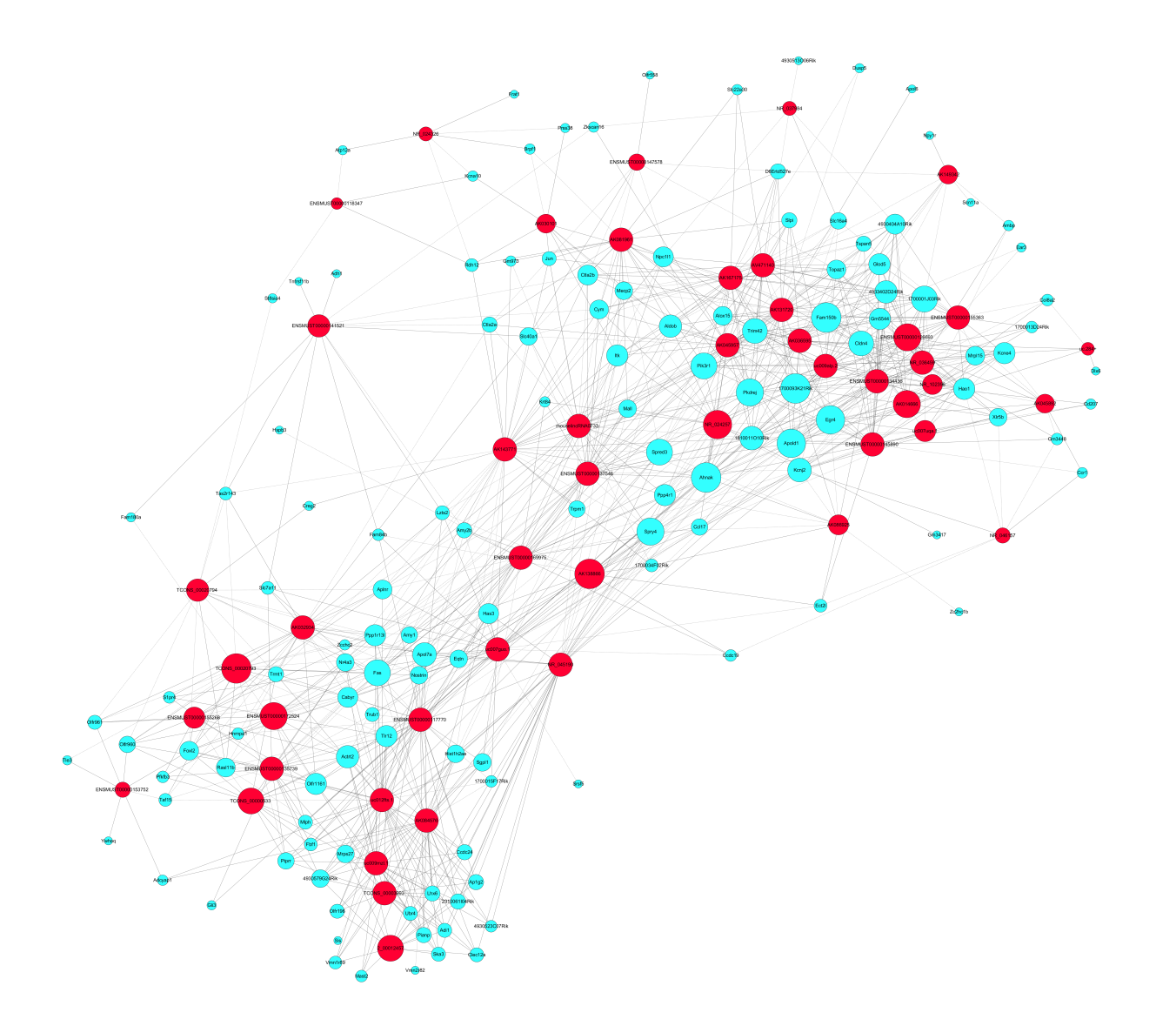
Supplementary Figure S3**. This Figure is the amplified Figure 4 (with higher resolution).

**
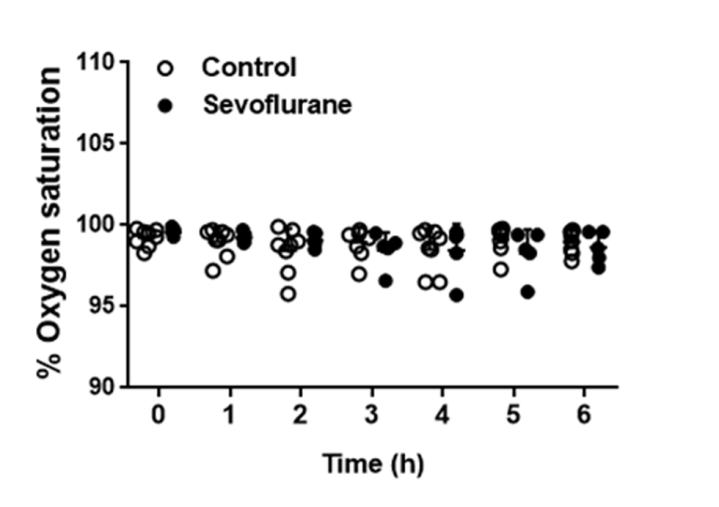
**

**Supplementary Figure S4**. Sevoflurane exposure does not alter oxygen saturation. Oxygen saturation was measured with MouseOx Plus Pulse Oximeter after 1 to 6 hours of 3% sevoflurane exposure in mice. n=5-8.

**Supplementary Tables**

**Supplementary Table S1**. Literature summary of sevoflurane-induced dysregulated mRNAs that are previously reported to be relevant to neurodevelopment in health and disorders

**Supplementary Table S2**. The bioinformatics analysis of predicted neurological diseases and functions of the sevoflurane-induced dysregulated mRNA profiles analyzed using Ingenuity Pathway Analysis software

**Supplementary Table S3**. The full gene name of sevoflurane-induced dysregulated mRNAs and lncRNAs

**Supplementary Table S4**. Sevoflurane-dysregulated highly correlated lncRNA-mRNA interaction pairs (absolute Pearson correlation coefficient above 0.9)

**Supplementary Table S5.** The bioinformatics analysis of co-expressed sevoflurane-dysregulated lncRNA and mRNAs involved in apoptosis and necrosis signaling networks

**Supplementary Table S6**. Sequence information for primers
