# Supplementary material for: Expression Signature of lncRNAs and mRNAs in Sevoflurane-Induced Mouse Brain Injury: Implication of Involvement of Wide Molecular Networks and Pathways
Source: Int J Mol Sci. 2021 Jan 30;22(3):1389. doi: 10.3390/ijms22031389 (PMC7869012; doi:10.3390/ijms22031389)
Supplement: Supplementary file 1 [file ijms-22-01389-s001.zip › ijms-1088385-supplementary/3. Supplementary Table S6 10.11.docx]

Supplementary Table 1. Sequence information for primers

| **Gene name**  **(Accession No.)** | **Sequence (from 5’ to 3’)** | **PCR Product length (bp)** |
| --- | --- | --- |
| Egr4 | F: GCTGCCTGCTAGGGACGCTC | 110 |
| NCBI GenBank NM_020596 | R: GGAAGCAGGAGTCTGTTAAGTCC |  |
| Slc40a1  NCBI GenBank NM_016917 | F: TGGAACTCTATGGAAACAGCCT | 111 |
|  | R: TGGCATTCTTATCCACCCAGT |  |
| Apold1  GenBank NM_001109914 | F: CGCTTCCAAGGATTGCTGC | 176 |
|  | R: CTGAGTGACAACCCCACGAT |  |
| 1700093K21Rik  NCBI GenBank NM_001110133 | F: ATGTTTACGCAAAGTGACACTGG | 171 |
|  | R: TTCATGGTTGTGACCTCTGTAAC |  |
| lncRNA Ak032553  NCBI Genbank Ak032553 | F: gccattttgcttcactcctc | 205 |
|  | R: tgacaccttctggtgacagc |  |
| lncRNA Ak134642  NCBI Genbank Ak134642 | F: gacagccaaagcagttgtca | 183 |
|  | R: atggatggaagcctgcatag |  |
| lncRNA Gm11525  Ensembl ENSMUST00000134436 | F: CACCCCAGAAGAGGGTATCA | 237 |
|  | R: ACCTTTAATCCCGGATGACC |  |
| lncRNA Foxp4  Ensembl ENSMUST00000153752 | F: TCTGCATCGGAGACAATCAG | 208 |
|  | R: GTTGCTGTTGGAAGTGCAGA |  |
| Actb  NCBI GenBank NM_007393.1 | F: AAGAGCTATGAGCTGCCTGA | 160 |
|  | R: TACGGATGTCAACGTCACAC |  |

Note: F: forward primer; R: reverse primer. The full gene names of the sevoflurane-induced dysregulated mRNAs and lncRNAs are detailed in Supplementary Table 6.
