# Supplementary material for: Expression Signature of lncRNAs and mRNAs in Sevoflurane-Induced Mouse Brain Injury: Implication of Involvement of Wide Molecular Networks and Pathways
Source: Int J Mol Sci. 2021 Jan 30;22(3):1389. doi: 10.3390/ijms22031389 (PMC7869012; doi:10.3390/ijms22031389)
Supplement: Supplementary file 1 [file ijms-22-01389-s001.zip › ijms-1088385-supplementary/4. Supplementary Table S1 10.11.docx]

| Supplementary Table 2. Literature summary of available information on the sevoflurane-induced. dysregulated mRNAs relevant to nervous system in health and disorders. | | | | |
| --- | --- | --- | --- | --- |
| **Gene Symbol** | **Role in neuro/brain development** | **Animal or cell models** | | **References** |
| Gm11992 | unknown | - | | - |
| Slpi | SLPI is necessary and sufficient for axon regeneration in the CNS. | This is a review | | (Carmel et al., 2015) |
|  | SLPI is identified as potential genetic amyotrophic lateral sclerosis (ALS, a fatal neurodegenerative disorder of motor neurons) biomarkers in human bone marrow mesenchymal stem cells obtained from sALS patients. | ALS transgenic mSOD1^G93A^ mouse | | (Lilo et al., 2013) |
|  | SLPI plays a pro-regenerative role and could potentially benefit the spinal cord injury. | This is a review | | (Siddiq and Hannila, 2015) |
| Pik3r1 | unknown | - | | - |
| Trpm1 | Trpm1 mRNA in retinas progressively increases from birth to PD21. Beside, localization of the TRPM1 in the mouse retina is plastic, modulated by use-dependence and availability of sustained excitatory input. | PD21 mice | | (Krizaj et al., 2010) |
|  | TRPM1 channel opening is essential for rod bipolar pathway establishment during development. | Mice | | (Kozuka et al., 2017) |
|  | TRPM1 recessive mutations abrogate ON bipolar cell function and could cause night blindness | patients | | (Li et al., 2009) |
| Bpifb9b | unknown | - | | - |
| Krt84 | unknown | - | | - |
| Cd207 | unknown | - | | - |
| 1700034F02Rik | unknown | - | | - |
| Amy2b | unknown | - | | - |
| Kcne4 | KCNE4 has a drastic inhibitory effect on currents generated by Kv1.1 and Kv1.3 potassium channels. | HEK293 cells | | (Grunnet et al., 2003) |
| 1700001J03Rik | unknown | - | | - |
| Glod5 | unknown | - | | - |
| Rasl11b | Rasl11b appeared only down-regulated in the HD82Q (mice model for Huntington's disease) hippocampus at late stages of the pathology. | mouse model N171-82Q (HD82Q mice) for Huntington's disease (HD) | | (Guiretti et al., 2016) |
| Slc22a30 | unknown | - | | - |
| Gm5544 | unknown | - | | - |
| Trub1 | unknown | - | | - |
| Alox15 | 15-lipoxygenase (ALOX15) catalyzes the conversion of docosa-hexaneoic acid into neuroprotectin D1 , hence is very essential in neurotrophic support and neuroprotection for the CNS. | Human retinal pigment epithelium cell-19 | | (Bazan, 2009) |
|  | 12/15-lipoxygenase (encoded by Alox15) modulates endogenous tau metabolism which is strongly implicated in Alzheimer disease (AD) pathogenesis. | Tg2576 mice | | (Giannopoulos et al., 2013) |
|  | 12/15-lipoxygenase (encoded by Alox15) is a key mediator of oxidative stress, contributing to neuronal cell death. | Samples were taken from two patients with ischemic stroke | | (Jung et al., 2015) |
|  | Alox15 deficient mice display increased vulnerability to 3-nitropropionic acid neurotoxicity. | Alox15^-/-^ mice | | (He et al., 2017) |
|  | 12/15-LOX (encoded by Alox15) is the central executioner in an oxidative stress-related neuronal death program. | HT22 cells (mouse hippocampal neuron cell line)  Primary neurons from  E16 ALOX15^-/-^ mice | | (Pallast et al., 2009) |
|  | Increased 12/15-LOX (encoded by Alox15) contribute to apoptotic pathways following global cerebral ischemia. | ALOX15 ^-/-^ mice | | (Yigitkanli et al., 2017) |
|  | 12/15-LOX inhibitors could rescue both neuronal as well as oligodendroglial cells from cell death induced by oxidative stress. | HT22 cell | | (van Leyen et al., 2008) |
| Dok2 | unknown | - | | - |
| Kcna10 | unknown | - | | - |
| Pkp1 | unknown | - | | - |
| Trim42 | unknown | - | | - |
| Cldn4 | Claudin 4 (Cldn4) has been found in mouse taste epithelium, with high abundance around the taste pore. | Wild type mice | | (Michlig et al., 2007) |
| Gm3417 | unknown | - | | - |
| Ctla2b | unknown | - | | - |
| Jun | One of multiple acute effects from ethanol on cerebellar granule neurons (CGNs) during neurodevelopment is an increased c-JUN and phosphorylated c-JUN protein, as well as c-jun mRNA. | CGNs isolated from PD3 SD rats | | (Acquaah-Mensah et al., 2001) |
|  | In animal model of schizophrenia, ketamine may evoke its stimulating effect on neurogenesis via a block of the  N-methyl-D-aspartate receptor directly by reducing the c-Fos/c-Jun expression. | SD rats | | (Keilhoff et al., 2004) |
| Prss38 | unknown | - | | - |
| Ly6c1 | unknown | - | | - |
| 4930404A10Rik | unknown | - | | - |
| 4930513O06Rik | unknown | - | | - |
| 1810011O10Rik | unknown | - | | - |
| Cabyr | unknown | - | | - |
| Hao1 | unknown | - | | - |
| Creg2 | unknown | - | | - |
| 4933402D24Rik | unknown | - | | - |
| Nostrin | unknown | - | | - |
| Cym | unknown | - | | - |
| Itk | unknown | - | | - |
| Taf15 | unknown | - | | - |
| Chst3 | unknown | - | | - |
| Xlr5b | unknown | - | | - |
| Scn11a | Nav1.9 channels (coded by Scn11a) are expressed in layer II and V medial prefrontal cortex (mPFC) pyramidal neurons and that its protein expression decreases during development. | rats | | (Gawlak et al., 2017) |
|  | gain-of-function mutations in SCN11A can be causative of an autosomal-dominant episodic pain disorder | Patients: families with autosomal-dominant episodic pain  Cell: mouse dorsal root ganglion (DRG) neurons | | (Leng et al., 2017) |
| 4933408B17Rik | unknown | - | | - |
| Hnrnpa1 | hnRNP-A1 is disregulated in AD patients, and its SNP rs7967622 C/C genotype is likely a risk factor for frontotemporal lobar degeneration (FTLD) in male populations. | 274 Italian patients with FTLD | | (Villa et al., 2011) |
|  | The autoimmune response of multiple sclerosis (MS) patients to hnRNP-A1 might contribute to its neurodegeneration. | Sera from MS patients  NT-2 and SK-N-SH cells | | (Lee et al., 2011) |
| 1700093K21Rik | unknown | - | | - |
| D6Ertd527e | unknown | - | | - |
| Pkdrej | unknown | - | | - |
| Vmn2r82 | unknown | - | | - |
| Srsf5 | Knockdown and overexpression studies reveal that SRSF5 can inhibit exon 7 inclusion. Its knockdown in cell lines derived from spinal muscular atrophy (SMA) patients could increase SMN2 exon 7 inclusion and hence is promising for therapy. | Cell model: human fibroblast cell line derived from a type I SMA patient with one copy of SMN2 | | (Wee et al., 2014) |
| Dgcr14 | Absence of Dgcr14 in the inter-peduncular nucleus results in altered regulation of rapid eye movement. | Gscl^− / −^ mice | | (Funato et al., 2010) |
|  | The human DGCR14 promoter polymorphism is a major risk factor for schizophrenia. | 235 trios composed of healthy parents and their affected offspring with schizophrenia. | | (Wang et al., 2006) |
| Ctla2a | Ctla2a is down-regulated in the cervical spinal cord after acupuncture at the acupoints GB34 and LR3 in the MPTP-induced parkinsonism model. | Animal model: C57BL/6 mice with MPTP-induced parkinsonism | | (Choi et al., 2011) |
| Sis | unknown | - | | - |
| Eqtn | unknown | - | | - |
| Apold1 | unknown | - | | - |
| Gm3448 | unknown | - | | - |
| Topaz1 | unknown | - | | - |
| Cabyr | unknown | - | | - |
| Gm973 | unknown | - | | - |
| 1700013D24Rik | unknown | - | | - |
| Amy1 | unknown | - | | - |
| Npc1l1 | Intranasal administration of Ezetimibe, a NPC1L1 pharmacological inhibitor leads to neuroprotection through autophagy activation after middle cerebral artery occlusion (MCAO) in rats. | Animal model: rat model of MCAO | | (Yu et al., 2018) |
| Ces1f | unknown | - | | - |
| Ahnak | nerve growth factor can increase the enlargeosome maker, Ahnak. | PC12 cell and SH-SY5Y | | (Schulte et al., 2010) |
|  | Co-IP assay identified Ahnak as binding protein with the hungtingtin (htt), hence considered to play crucial role in HD. | C57BL/6 adult mice | | (Yao et al., 2014) |
|  | Ahnak deficiency promotes hippocampal neurogenesis in adult mice. | Ahnak deficient mice at 12 weeks of age | | (Shin et al., 2015) |
|  | Constitutive Ahnak KO mice or forebrain glutamatergic neuron-selective Ahnak KO mice develop a depression-like behavioral phenotype. | Ahnak KO mice | | (Jin， et al., 2019) |
| Ambp | AMBP-1 could elevate cAMP level, activate PKA, and protect neural cells from hypoxia induced apoptosis. | human neuroblastoma cell line SH-SY5Y | | (Wang and Yang, 2009) |
|  | Treatment of human AMBP-1 reduced  neuron apoptosis and morphological damage, inhibited neutrophil infiltration in the brain, and hence can reduce stroke-induced brain injury in rats. | stroke-induced brain injury in rats | | (Chaung et al., 2011) |
| Zkscan16 | unknown | - | | - |
| Ect2l | unknown | - | | - |
| Olfr691 | Olfr691senses a wide range of both short and medium chain fatty acids, binding to carboxylic acids with carbon lengths of three (propionate) to eight (octanoate). | Animal model: C57Bl/6 kidney | | (Rajkumar et al., 2014) |
| Fas | unknown | - | | - |
| 1700015F17Rik | unknown | - | | - |
| Fer1l4 | unknown | - | | - |
| Rdh12 | unknown | - | | - |
| Aldob | unknown | - | | - |
| Ccdc19 | unknown | - | | - |
| Apol6 | unknown | - | | - |
| Dlx6 | unknown | - | | - |
| Ccl17 | unknown | - | | - |
| Mall | unknown | - | | - |
| 4930523C07Rik | unknown | - | | - |
| Ear3 | unknown | - | | - |
| Gli3 | Neurological injury related GLI3 is differentially-regulated in amniotic fluid supernatant of fetuses with myelomeningocele. | Ten pregnant women undergoing fetal surgery for myelomeningocele. | | (Tarui et al., 2017) |
| Ifi44 | unknown | - | | - |
| Ppp1r13l | unknown | - | | - |
| Fam84b | Fam84b gene expression demarcates a unique region of the right habenula that is the site of innervation by olfactory axons. | zebrafish | | (deCarvalho et al., 2013) |
| Apol7a | unknown | - | | - |
| Clec12a | unknown | - | | - |
| Tas2r143 | TAS2R143 was validated as a chemical danger receptor transducing both alarm pheromone and predator-derived kairomone signals. | Cell model: Grueneberg ganglion from adult mice | | (Moine et al., 2018) |
| Adh1 | unknown | - | | - |
| Fam180a | unknown | - | | - |
| Ap1g2 | unknown | - | | - |
| Fam150b | unknown | - | | - |
| Adcyap1 | unknown | - | | - |
| Slc7a11 | In amyotrophic lateral sclerosis mice, Slc7a11 deletion led to earlier symptom onset but a significantly slowed progressive disease phase, which resulted in more surviving motor neurons. | amyotrophic lateral sclerosis mice | | (Mesci et al., 2015) |
|  | Lysine-specific demethylase 1 inhibitors protected spiral ganglion neurons from cisplatin-induced neuron loss by increasing the antioxidant gene Slc7a11 expression. | cochlear explants from PD3 mice | | (Li et al., 2015) |
|  | Slc7a11 is present in select blood/brain/CSF interface areas and in an astrocyte subpopulation. | adult C57BL6 mice | | (Ottestad-Hansen et al., 2018) |
|  | Dysregulated ferroptosis is strongly implicated in neurotoxicity and neurodegenerative diseases.  NADPH oxidase promotes ferroptosis by promotion of ROS production and inhibition of SLC7A11 expression. | - | | (Xie et al., 2016) |
|  | in vivo propagated and intracranially implanted SLC7A11-expressing  tumors produced peritumoral glutamate excitotoxicity and induced seizures. | Tumor-implanted mice | | (Robert et al., 2015) |
| Egr4 | Egr4 is required for the formation of the brain primordia and head regeneration | planarians | | (Fraguas et al., 2014) |
|  | Egr4 upregulation of could demote the expression of pro-inflammatory cytokines in MCAO rats as protector for ischemic stroke. | Rat middle cerebral artery occlusion (MAO) model | | (Niu et al., 2018) |
|  | Egr4 serve as important component during BDNF-dependent KCC2 gene regulation in immature neurons. | Primary hippocampal cultures from E17 mice | | (Ludwig et al., 2011) |
|  | EGR-4 contributes to the effects of morphine on neuronal plasticity. | F11 cells ( cell model for DRG  neurons) and DRG neurons from E18 SD rats | | (Rothe et al., 2012) |
|  | Early rotary non-uniform magnetic fields therapy produced significant benefit in central nervous regeneration, through up-regulating EGR4. | planarian | | [26(Chen et al., 2016) |
| 2310061I04Rik | unknown | - | | - |
| Brpf1 | unknown | - | | - |
| St8sia4 | St8sia4 deficiency could reduce interneuron densities in mPFC and loss of polySia synthesizing capacity. | St8sia4^-/-^ mice | | (Krocher et al., 2014) |
|  | St8sia4 expression in the embryonic and adult nervous system is often confined to regions  of neuronal migration. | Developing and adult zebrafish | | (Rieger et al., 2008) |
|  | ST8SIA4 delays oligodendrocyte differentiation. | St8sia4^-/-^ | | (Werneburg et al., 2017) |
| Mecp2 | MECP2 is identified as the major causative gene of Rett syndrome (an X-linked neurodevelopmental disorder). | This is a review | | (Gold et al., 2018) |
|  | The absence of MECP2 could lead to altered mitochondrial function and elevated levels of cellular oxidative stress in Rett syndrome patients. | This is a review | | (Shulyakova et al., 2017) |
|  | Prenatally exposed EtOH induces hyperactive, inattentive and impulsive behaviors in rodent offspring that might be related to global epigenetic changes including the signiﬁcantly decreased the expression of MECP2 in both prefrontal cortex and striatum. | Female ICR mice and Sprague–Dawley rats | | (Kim et al., 2013) |
|  | MECP2 phosphorylation induces a direct interaction with DGCR8, leading to reduced microRNA processing and retardation of dendritic growth. | MeCP2-depleted cells | | (Cheng et al., 2014; Woo and Kim, 2014) |
|  | MECP2 serve as a maintenance factor in the adult hippocampus that preserves signal responsiveness of the genome and allows for integrity of cognitive functions | male C57BL/6N mice | | (Karaca et al., 2018) |
|  | Mild overexpression of Mecp2 in mice could cause a higher susceptibility toward seizures. | Mecp2^WT_EGFP^ transgenic (TG) mouse | | (Bodda et al., 2013) |
|  | Loss-of-function mutation in Mecp2 causes impaired E/I balance onto CA3 pyramidal neurons, leading to a hyperactive hippocampal network, likely contributing to limbic seizures. | Mecp2 knockout male mice (referred to as Mecp2^-/y^) | | (Calfa et al., 2015) |
|  | MeCP2 as “reader” of DNA methylation is important in neurodevelopment and neuroplasticity. | This is a review | | (Fasolino and Zhou, 2017) |
|  | MECP2 is regulated post-transcriptionally during in vitro differentiation of human embryonic stem cells (hESCs) into cortical neurons. | H9 hESCs | | (Rodrigues et al., 2016) |
|  | MECP2 controls the excitatory presynaptic function through regulation of gene expression. | MECP2 null knockout mice | | (Nelson et al., 2006) |
|  | Decreased dopamine transmission due to heterogeneous Mecp2 expression contributes to the Parkinsonian features in Mecp2^+/−^ mice. | Mecp2^+/−^ mice | | (Gantz et al., 2011) |
|  | Impaired methylation and mutations of mecp2 have been associated with autistic spectrum disorders. | This is a review | | (Currenti, 2010) |
|  | MeCP2 regulates global histone modifications during a critical postnatal  stage of neuronal maturation. | Wildtype C57BL/6J and Mecp2^tm1.1Bird/y^ tissue | | (Thatcher and LaSalle, 2006) |
| Frat1 | unknown | - | | - |
| Atp12a | Loss of ATP13A2 function participates in mitochondrial maintenance and oxidative stress which are centrally implicated in Parkinson’s disease. | Cell model: primary mouse cortical neurons and SH-SY5Y cells ( human neuroblastoma cell line) | | (Gusdon et al., 2012) |
| Ptprr | Ptprr^-/-^ mice display motor coordination defects. | Wild type and Ptprr^-/-^ mice | | (Chirivi et al., 2007; Schmitt et al., 2009; Chesini et al., 2011) |
|  | PTPRR over-expression in the hippocampal DG could lead to more neuron apoptosis, less cell proliferation, depression onset and increased sensitivity to chronic mild stress | C57 BL/6J mice | | (Li et al., 2016) |
|  | long-term depression in the cerebellum of Ptprr ^−/−^ mice is strongly impeded. | Ptprr ^−/−^ mice | | (Erkens et al., 2015) |
| 4930579G24 | unknown | - | | - |
| Rik | unknown | - | | - |
| Tlr12 | Tlr12 was abundant in neurons, but not in astrocytes and ependymal cells, and parasite infection increased its protein expression at 3 weeks post injection in the whole brain. | Animal model: mouse neurocysticercosis model (M. corti parasites treated Balb/c mice) | | (Mishra et al., 2008) |
| Ppp4r1 | unknown | - | | - |
| Mrpl15 | unknown | - | | - |
| Trmt1 | unknown | - | | - |
| Zcchc2 | unknown | - | | - |
| Ubr4 | Ubr4 (p600) work as a critical protein in the mammalian brain with roles in neurogenesis, neuronal migration, neuronal signaling and survival.  (Reports about Ubr4 in neurodevelopment is too many, hence we choose this review article to describe its general role.) | | | (Parsons et al., 2015) |
| Ska3 | Increased extracellular glucocorticoid levels signiﬁcantly reduce SKA3 expression levels, followed by altered neuronal cell viability and neurite development. | | Cell model: PC12 cells (rat pheochromocytoma cell line) treated with glucocorticoid | (Zhao et al., 2018) |
|  | SKA3 level positively regulates neurite outgrowth. | | Human neuroblastoma IMR-32 cells | (Tong et al., 2013) |
| Ywhaq | unknown | | - | - |
| Actrt2 | unknown | | - | - |
| Tspan5 | The level of Tspan-5 mRNA expression correlates very well with the differentiation status of particular neurons in the Cerebellum. Suggesting it might be important for distinct phases of neuronal maturation. | | Normal and L7En-2 Transgenic Mice | (Juenger et al., 2008) |
| Aplnr | unknown | | - | - |
| Ccdc24 | unknown | | - | - |
| Dusp5 | unknown | | - | - |
| Foxl2 | unknown | | - | - |
| Kcnj2 | unknown | | - | - |
| Lhx6 | The transcription factor Lhx6 is specific for postmitotic cortical interneurons generated in the medial ganglionic eminence. | | C57BL/6 mice | (Steinecke et al., 2014) |
| Igfbp5 | unknown | | - | - |
| Olfr1161 | unknown | | - | - |
| Olfr961 | Olfr961 was validated as one of the eugenol-responsive odorant receptors. | | Animal model:  S100a5–tauGFP mouse treated with  eugenol  Cell model: Hana 3A cells treated with eugenol | (McClintock et al., 2014) |
| Zc2hc1b | unknown | | - | - |
| Slc16a4 | unknown | | - | - |
| Lzts2 | unknown | | - | - |
| Pianp | The lack of PIANP impairs GABA B receptors-mediated inhibition of excitatory postsynaptic currents at CA3/CA1 synapses. | | Animal model: Pianp^−/−^ mice in the C57BL/6 background | (Dinamarca et al., 2019) |
| Olfr558 | Olfr558 serves as a microbial metabolite sensor in the neural sensory pathways. | | Cell model: enterochromaffin cell (an intestinal endocrine cell) | (Bellono et al., 2017) |
| Mrps27 | unknown | | - | - |
| Nr4a3 | unknown | | - | - |
| Slc40a1 | unknown | | - | - |
| Has3 | Has3 knock-out causes altered neuronal activity and seizures via reduction in brain extracellular space. | | Has3 knock-out mice | (Arranz et al., 2014) |
| Hspb3 | unknown | | - | - |
| Sgpl1 | Neural-targeted depletion of SGPL1 causes cognitive deﬁcits and a decrease of brain phosphatidylethanolamine content along with impaired autophagy and a consequent accumulation of neurodegenerative biomarkers in the brains of SGPL1^ﬂ/ﬂ/Nes^ mice. | | Animal model: SGPL1^ﬂ/ﬂ/Nes^ mice | (Mitroi et al., 2017) |
|  | SGPL1 mutations as a cause of a distinct form of Charcot-Marie-Tooth disease (a heterogeneous group of peripheral neuropathis). | | Charcot-Marie-Tooth disease patients | (Atkinson et al., 2017) |
| Vmn1r89 | unknown | | - | - |
| Spred3 | unknown | | - | - |
| Pfkfb3 | unknown | | - | - |
| Adi1 | unknown | | - | - |
| Mlph | unknown | | - | - |
| Col8a2 | unknown | | - | - |
| Olfr960 | Olfr960 was validated as one of the eugenol-responsive odorant receptors. | | Animal model:  S100a5–tauGFP mouse treated with  eugenol  Cell model: Hana 3A cells treated with eugenol | (McClintock et al., 2014) |
| Tle3 | unknown | | - | - |
| S1pr4 | S1pr4 is expressed in neurons at later stages (since E9.5) during mouse embryonic development. | | Animal model: mouse embryo | (Meng and Lee, 2009) |
| Olfr196 | unknown | | - | - |
| Tnfrsf11b | unknown | | - | - |
| Npy1r | Overexpression of MECP2_e1 (responsible for Rett syndrome) during neurodevelopment could lead to upregulation of NPY1R. | | human neuroblastoma cell line SK-N-SH was differentiated into neurons | (Orlic-Milacic et al., 2014) |
| Fbf1 | unknown | | - | - |
| Spry4 | unknown | | - | - |
| Hist1h2ae | unknown | | - | - |
| Ccr1 | unknown | | - | - |
| Mast2 | In *Mast1* Leu278del mice, Mast2 and Mast3 levels are diminished, and the mice have an enlarged corpus callosum and smaller cerebellum. Hence mutation of the Mast family might contribute to the neurodevelopmental diseases. | | Patients: mega-corpus-callosum syndrome with cerebellar hypoplasia and cortical malformations patients  Animal model: *Mast1* Leu278del mice | (Tripathy et al., 2018) |
|  | Neuronal survival (a signature of rabies virus virulence) requires the selective association of the PDZ-BS  of G with the PDZ domains of MAST1 And MAST2. | | Cell model: Laboratory strains of rabies virus (RABV) infected human neuroblastoma cell lines SK-N-SH and a subclone of SH-SY5Y  Animal model: rRABVs administrated mice | (Prehaud et al., 2010) |
|  | MAST2 function as negative regulators of cell survival pathways, and its silencing promotes neuronal survival. | | Cell model: In human neuroblastoma cells infected with RABV | (Terrien et al., 2012) |
|  | MAST2 could inhibit the neurite outgrowth and lysophosphatidic acid-induced neurite retraction. | | Cell model：Primary CGN culture from 7-day-old Wistar rat pups | (Loh et al., 2008) |

*Note: when the reports of particular gene are too many, we choose to summarize using review article rather than displaying all original articles. AD, Alzheimer disease; ALOX15, 15-lipoxygenase; ALS, amyotrophic lateral sclerosis; CGN, cerebellar granule neurons; Cldn4, Claudin 4; DRG, dorsal root ganglion; FTLD, frontotemporal lobar degeneration; HD, Huntington's disease; hESCs, human embryonic stem cells; MCAO, middle cerebral artery occlusion; mPFC, medial prefrontal cortex; MS, multiple sclerosis; PD, postnatal day; RABV, rabies virus; SMA, spinal muscular atrophy. The full gene names of the sevoflurane-induced dysregulated mRNAs are detailed in Supplementary Table 6.*

**References**

Acquaah-Mensah GK, Leslie SW, Kehrer JP (2001) Acute exposure of cerebellar granule neurons to ethanol suppresses stress-activated protein kinase-1 and concomitantly induces AP-1. Toxicol Appl Pharm 175:10-18.

Arranz AM, Perkins KL, Irie F, Lewis DP, Hrabe J, Xiao F, Itano N, Kimata K, Hrabetova S, Yamaguchi Y (2014) Hyaluronan deficiency due to Has3 knock-out causes altered neuronal activity and seizures via reduction in brain extracellular space. J Neurosci 34:6164-6176.

Atkinson D, Glumac JN, Asselbergh B, Ermanoska B, Blocquel D, Steiner R, Estrada-Cuzcano A, Peeters K, Ooms T, De Vriendt E, Yang XL, Hornemann T, Rasic VM, Jordanova A (2017) Sphingosine 1-phosphate lyase deficiency causes Charcot-Marie-Tooth neuropathy. Neurology 88:533-542.

Bazan NG (2009) Cellular and molecular events mediated by docosahexaenoic acid-derived neuroprotectin D1 signaling in photoreceptor cell survival and brain protection. Prostaglandins, leukotrienes, and essential fatty acids 81:205-211.

Bellono NW, Bayrer JR, Leitch DB, Castro J, Zhang CC, O'Donnell TA, Brierley SM, Ingraham HA, Julius D (2017) Enterochromaffin Cells Are Gut Chemosensors that Couple to Sensory Neural Pathways. Cell 170.

Bodda C, Tantra M, Mollajew R, Arunachalam JP, Laccone FA, Can K, Rosenberger A, Mironov SL, Ehrenreich H, Mannan AU (2013) Mild Overexpression of Mecp2 in Mice Causes a Higher Susceptibility toward Seizures. Am J Pathol 183:195-210.

Calfa G, Li W, Rutherford JM, Pozzo-Miller L (2015) Excitation/Inhibition Imbalance and Impaired Synaptic Inhibition in Hippocampal Area CA3 of Mecp2 Knockout Mice. Hippocampus 25:159-168.

Carmel JB, Young W, Hart RP (2015) Flipping the transcriptional switch from myelin inhibition to axon growth in the CNS. Front Mol Neurosci 8.

Chaung WW, Wu RQ, Ji YX, Wang ZM, Dong WF, Cheyuo C, Qi L, Qiang XL, Wang HC, Wang P (2011) Peripheral Administration of Human Adrenomedullin and Its Binding Protein Attenuates Stroke-Induced Apoptosis and Brain Injury in Rats. Mol Med 17:1075-1083.

Chen Q, Lin GM, Wu N, Tang SW, Zheng ZJ, Lin MCM, Xu GX, Liu H, Deng YY, Zhang XY, Chen SP, Wang XM, Niu HB (2016) Early exposure of rotating magnetic fields promotes central nervous regeneration in planarian Girardia sinensis. Bioelectromagnetics 37:244-255.

Cheng TL, Wang ZZ, Liao QM, Zhu Y, Zhou WH, Xu WQ, Qiu ZL (2014) MeCP2 Suppresses Nuclear MicroRNA Processing and Dendritic Growth by Regulating the DGCR8/Drosha Complex. Dev Cell 28:547-560.

Chesini IM, Debyser G, Croes H, ten Dam GB, Devreese B, Stoker AW, Hendriks WJAJ (2011) PTPBR7 Binding Proteins in Myelinating Neurons of the Mouse Brain. Int J Biol Sci 7:978-991.

Chirivi RGS, Noordman YE, Van der Zee CEEM, Hendriks WJAJ (2007) Altered MAP kinase phosphorylation and impaired motor coordination in PTPRR deficient mice. J Neurochem 101:829-840.

Choi YG, Yeo S, Hong YM, Kim SH, Lim S (2011) Changes of gene expression profiles in the cervical spinal cord by acupuncture in an MPTP-intoxicated mouse model: Microarray analysis. Gene 481:7-16.

Currenti SA (2010) Understanding and Determining the Etiology of Autism. Cell Mol Neurobiol 30:161-171.

deCarvalho TN, Akitake CM, Thisse C, Thisse B, Halpern ME (2013) Aversive cues fail to activate fos expression in the asymmetric olfactory-habenula pathway of zebrafish. Front Neural Circuit 7.

Dinamarca MC, Raveh A, Schneider A, Fritzius T, Fruh S, Rem PD, Stawarski M, Lalanne T, Turecek R, Choo M, Besseyrias V, Bildl W, Bentrop D, Staufenbiel M, Gassmann M, Fakler B, Schwenk J, Bettler B (2019) Complex formation of APP with GABA(B) receptors links axonal trafficking to amyloidogenic processing. Nat Commun 10.

Erkens M, Tanaka-Yamamoto K, Cheron G, Marquez-Ruiz J, Prigogine C, Schepens JTG, Kasri NN, Augustine GJ, Hendriks WJAJ (2015) Protein tyrosine phosphatase receptor type R is required for Purkinje cell responsiveness in cerebellar long-term depression. Mol Brain 8.

Fasolino M, Zhou ZL (2017) The Crucial Role of DNA Methylation and MeCP2 in Neuronal Function. Genes-Basel 8.

Fraguas S, Barberan S, Iglesias M, Rodriguez-Esteban G, Cebria F (2014) egr-4, a target of EGFR signaling, is required for the formation of the brain primordia and head regeneration in planarians. Development 141:1835-1847.

Funato H, Sato M, Sinton CM, Gautron L, Williams SC, Skach A, Elmquist JK, Skoultchi AI, Yanagisawa M (2010) Loss of Goosecoid-like and DiGeorge syndrome critical region 14 in interpeduncular nucleus results in altered regulation of rapid eye movement sleep. P Natl Acad Sci USA 107:18155-18160.

Gantz SC, Ford CP, Neve KA, Williams JT (2011) Loss of Mecp2 in Substantia Nigra Dopamine Neurons Compromises the Nigrostriatal Pathway. J Neurosci 31:12629-12637.

Gawlak M, Szulczyk B, Berlowski A, Grzelka K, Stachurska A, Pelka J, Czarzasta K, Malecki M, Kurowski P, Nurowska E, Szulczyk P (2017) Age-dependent expression of Nav1.9 channels in medial prefrontal cortex pyramidal neurons in rats. Dev Neurobiol 77:1371-1384.

Giannopoulos PF, Joshi YB, Chu J, Pratico D (2013) The 12-15-lipoxygenase is a modulator of Alzheimer's-related tau pathology in vivo. Aging Cell 12:1082-1090.

Gold WA, Krishnarajy R, Ellaway C, Christodoulou J (2018) Rett Syndrome: A Genetic Update and Clinical Review Focusing on Comorbidities. Acs Chem Neurosci 9:167-176.

Grunnet M, Rasmussen HB, Hay-Schmidt A, Rosenstierne M, Klaerke DA, Olesen SP, Jespersen T (2003) KCNE4 is an inhibitory subunit to Kv1.1 and Kv1.3 potassium channels. Biophys J 85:1525-1537.

Guiretti D, Sempere A, Lopez-Atalaya JP, Ferrer-Montiel A, Barco A, Valor LM (2016) Specific promoter deacetylation of histone H3 is conserved across mouse models of Huntington's disease in the absence of bulk changes. Neurobiol Dis 89:190-201.

Gusdon AM, Zhu JH, Van Houten B, Chu CT (2012) ATP13A2 regulates mitochondrial bioenergetics through macroautophagy. Neurobiol Dis 45:962-972.

He Y, Akumuo RC, Yang Y, Hewett SJ (2017) Mice deficient in L-12/15 lipoxygenase show increased vulnerability to 3-nitropropionic acid neurotoxicity. Neurosci Lett 643:65-69.

Jin， J, Bhatti， DL, Lee， K-W, Medrihan， L, Cheng， J, Wei， J, Zhong， P, Yan， Z, Kooiker， C, Song， C, Ahn， J-H, Obermair， GJ, Lee， A, Gresack， J, Greengard， P, Kim Y (2019) Ahnak scaffolds p11/Anxa2 complex and L-type voltage-gated calcium channel and modulates depressive behavior. Molecular Psychiatry.

Juenger H, Holst MI, Duffe K, Jankowski J, Baader SL (2008) Tetraspanin-5 (Tm4sf9) mRNA expression parallels neuronal maturation in the cerebellum of normal and L7En-2 transgenic mice (vol 483, pg 318, 2005). J Comp Neurol 508:676-676.

Jung JE, Karatas H, Liu Y, Yalcin A, Montaner J, Lo EH, van Leyen K (2015) STAT-dependent upregulation of 12/15-lipoxygenase contributes to neuronal injury after stroke. J Cerebr Blood F Met 35:2043-2051.

Karaca KG, Brito DVC, Zeuch B, Oliveira AMM (2018) Adult hippocampal MeCP2 preserves the genomic responsiveness to learning required for long-term memory formation. Neurobiol Learn Mem 149:84-97.

Keilhoff G, Bernstein HG, Becker A, Grecksch G, Wolf G (2004) Increased neurogenesis in a rat ketamine model of schizophrenia. Biol Psychiat 56:317-322.

Kim P, Park JH, Choi CS, Choi I, Joo SH, Kim MK, Kim SY, Kim KC, Park SH, Kwon KJ, Lee J, Han SH, Ryu JH, Cheong JH, Han JY, Ko KN, Shin CY (2013) Effects of Ethanol Exposure During Early Pregnancy in Hyperactive, Inattentive and Impulsive Behaviors and MeCP2 Expression in Rodent Offspring. Neurochem Res 38:620-631.

Kozuka T, Chaya T, Tamalu F, Shimada M, Fujimaki-Aoba K, Kuwahara R, Watanabe SI, Furukawa T (2017) The TRPM1 Channel Is Required for Development of the Rod ON Bipolar Cell-AII Amacrine Cell Pathway in the Retinal Circuit. J Neurosci 37:9889-9900.

Krizaj D, Huang W, Furukawa T, Punzo C, Xing W (2010) Plasticity of TRPM1 expression and localization in the wild type and degenerating mouse retina. Vision Res 50:2460-2465.

Krocher T, Rockle I, Diederichs U, Weinhold B, Burkhardt H, Yanagawa Y, Gerardy-Schahn R, Hildebrandt H (2014) A crucial role for polysialic acid in developmental interneuron migration and the establishment of interneuron densities in the mouse prefrontal cortex. Development 141:3022-3032.

Lee S, Xu LJ, Shin YJ, Gardner L, Hartzes A, Dohan FC, Raine C, Homayouni R, Levin MC (2011) A potential link between autoimmunity and neurodegeneration in immune-mediated neurological disease. J Neuroimmunol 235:56-69.

Leng XR, Qi XH, Zhou YT, Wang YP (2017) Gain-of-function mutation p.Arg225Cys in SCN11A causes familial episodic pain and contributes to essential tremor. J Hum Genet 62:641-646.

Li A, He YZ, Sun S, Cai CF, Li HW (2015) Lysine-specific demethylase 1 inhibitors protect cochlear spiral ganglion neurons against cisplatin-induced damage. Neuroreport 26:539-547.

Li XR, Liu ZF, Li W, Sun N, Xu Y, Xie ZC, Zhang KR (2016) PTPRR regulates ERK dephosphorylation in depression mice model. J Affect Disorders 193:233-241.

Li Z, Sergouniotis PI, Michaelides M, Mackay DS, Wright GA, Devery S, Moore AT, Holder GE, Robson AG, Webster AR (2009) Recessive Mutations of the Gene TRPM1 Abrogate ON Bipolar Cell Function and Cause Complete Congenital Stationary Night Blindness in Humans. Am J Hum Genet 85:711-719.

Lilo E, Wald-Altman S, Solmesky LJ, Ben Yaakov K, Gershoni-Emek N, Bulvik S, Kassis I, Karussis D, Perlson E, Weil M (2013) Characterization of human sporadic ALS biomarkers in the familial ALS transgenic mSOD1(G93A) mouse model. Hum Mol Genet 22:4720-4725.

Loh SHY, Francescut L, Lingor P, Bahr M, Nicotera P (2008) Identification of new kinase clusters required for neurite outgrowth and retraction by a loss-of-function RNA interference screen. Cell Death Differ 15:283-298.

Ludwig A, Uvarov P, Soni S, Thomas-Crusells J, Airaksinen MS, Rivera C (2011) Early Growth Response 4 Mediates BDNF Induction of Potassium Chloride Cotransporter 2 Transcription. J Neurosci 31:644-649.

McClintock TS, Adipietro K, Titlow WB, Breheny P, Walz A, Mombaerts P, Matsunami H (2014) In Vivo Identification of Eugenol-Responsive and Muscone-Responsive Mouse Odorant Receptors. J Neurosci 34:15669-15678.

Meng H, Lee VM (2009) Differential Expression of Sphingosine-1-Phosphate Receptors 1-5 in the Developing Nervous System. Dev Dynam 238:487-500.

Mesci P, Zaidi S, Lobsiger CS, Millecamps S, Escartin C, Seilhean D, Sato H, Mallat M, Boillee S (2015) System x(C)(-) is a mediator of microglial function and its deletion slows symptoms in amyotrophic lateral sclerosis mice. Brain 138:53-68.

Michlig S, Damak S, Le Coutre J (2007) Claudin-based permeability barriers in taste buds. J Comp Neurol 502:1003-1011.

Mishra BB, Gundra UM, Teale JM (2008) Expression and distribution of Toll-like receptors 11-13 in the brain during murine neurocysticercosis. J Neuroinflamm 5.

Mitroi DN, Karunakaran I, Graler M, Saba JD, Ehninger D, Ledesma MD, van Echten-Deckert G (2017) SGPL1 (sphingosine phosphate lyase 1) modulates neuronal autophagy via phosphatidylethanolamine production. Autophagy 13:885-899.

Moine F, Brechbuhl J, Tosato MN, Beaumann M, Broillet MC (2018) Alarm pheromone and kairomone detection via bitter taste receptors in the mouse Grueneberg ganglion. Bmc Biol 16.

Nelson ED, Kavalali ET, Monteggia LM (2006) MeCP2-dependent transcriptional repression regulates excitatory neurotransmission. Curr Biol 16:710-716.

Niu RN, Shang XP, Teng JF (2018) Overexpression of Egr2 and Egr4 protects rat brains against ischemic stroke by downregulating JNK signaling pathway. Biochimie 149:62-70.

Orlic-Milacic M, Kaufman L, Mikhailov A, Cheung AYL, Mahmood H, Ellis J, Gianakopoulos PJ, Minassian BA, Vincent JB (2014) Over-Expression of Either MECP2_e1 or MECP2_e2 in Neuronally Differentiated Cells Results in Different Patterns of Gene Expression. Plos One 9.

Ottestad-Hansen S, Hu QX, Follin-Arbelet VV, Bentea E, Sato H, Massie A, Zhou Y, Danbolt NC (2018) The cystine-glutamate exchanger (xCT, Slc7a11) is expressed in significant concentrations in a subpopulation of astrocytes in the mouse brain. Glia 66:951-970.

Pallast S, Arai K, Wang XY, Lo EH, van Leyen K (2009) 12/15-Lipoxygenase targets neuronal mitochondria under oxidative stress. J Neurochem 111:882-889.

Parsons K, Nakatani Y, Nguyen MD (2015) p600/UBR4 in the central nervous system. Cell Mol Life Sci 72:1149-1160.

Prehaud C, Wolff N, Terrien E, Lafage M, Megret F, Babault N, Cordier F, Tan GS, Maitrepierre E, Menager P, Chopy D, Hoos S, England P, Delepierre M, Schnell MJ, Buc H, Lafon M (2010) Attenuation of Rabies Virulence: Takeover by the Cytoplasmic Domain of Its Envelope Protein. Sci Signal 3.

Rajkumar P, Aisenberg WH, Acres OW, Protzko RJ, Pluznick JL (2014) Identification and Characterization of Novel Renal Sensory Receptors. Plos One 9.

Rieger S, Volkmann K, Koster RW (2008) Polysialyltransferase expression is linked to neuronal migration in the developing and adult zebrafish. Dev Dynam 237:276-285.

Robert SM, Buckingham SC, Campbell SL, Robel S, Holt KT, Ogunrinu-Babarinde T, Warren PP, White DM, Reid MA, Eschbacher JM, Berens ME, Lahti AC, Nabors LB, Sontheimer H (2015) SLC7A11 expression is associated with seizures and predicts poor survival in patients with malignant glioma. Sci Transl Med 7.

Rodrigues DC, Kim DS, Yang G, Zaslavsky K, Ha KCH, Mok RSF, Ross PJ, Zhao M, Piekna A, Wei W, Blencowe BJ, Morris Q, Ellis J (2016) MECP2 Is Post-transcriptionally Regulated during Human Neurodevelopment by Combinatorial Action of RNA-Binding Proteins and miRNAs. Cell Rep 17:720-734.

Rothe K, Solinski HJ, Boekhoff I, Gudermann T, Breit A (2012) Morphine Activates the E Twenty Six-Like Transcription Factor-1/Serum Response Factor Pathway via Extracellular Signal-Regulated Kinases 1/2 in F11 Cells Derived from Dorsal Root Ganglia Neurons. J Pharmacol Exp Ther 342:41-52.

Schmitt I, Bitoun E, Manto M (2009) PTPRR, Cerebellum, and Motor Coordination. Cerebellum 8:71-73.

Schulte C, Racchetti G, D'Alessandro R, Meldolesi J (2010) A New Form of Neurite Outgrowth Sustained by the Exocytosis of Enlargeosomes Expressed under the Control of REST. Traffic 11:1304-1314.

Shin JH, Kim YN, Kim IY, Choi DH, Yi SS, Seong JK (2015) Increased Cell Proliferations and Neurogenesis in the Hippocampal Dentate Gyrus of Ahnak Deficient Mice. Neurochem Res 40:1457-1462.

Shulyakova N, Andreazza AC, Mills LR, Eubanks JH (2017) Mitochondrial Dysfunction in the Pathogenesis of Rett Syndrome: Implications for Mitochondria-Targeted Therapies. Front Cell Neurosci 11.

Siddiq MM, Hannila SS (2015) Looking downstream: the role of cyclic AMP-regulated genes in axonal regeneration. Front Mol Neurosci 8.

Steinecke A, Gampe C, Nitzsche F, Bolz J (2014) DISCI knockdown impairs the tangential migration of cortical interneurons by affecting the actin cytoskeleton. Front Cell Neurosci 8.

Tarui T, Kim A, Flake A, McClain L, Stratigis JD, Fried I, Newman R, Slonim DK, Bianchi DW (2017) Amniotic fluid transcriptomics reflects novel disease mechanisms in fetuses with myelomeningocele. Am J Obstet Gynecol 217.

Terrien E, Chaffotte A, Lafage M, Khan Z, Prehaud C, Cordier F, Simenel C, Delepierre M, Buc H, Lafon M, Wolff N (2012) Interference with the PTEN-MAST2 Interaction by a Viral Protein Leads to Cellular Relocalization of PTEN. Sci Signal 5.

Thatcher KN, LaSalle JM (2006) Dynamic Changes in Histone H3 Lysine 9 Acetylation Localization Patterns During Neuronal Maturation Require MeCP2. Epigenetics-Us 1:24-31.

Tong CW, Wang JL, Jiang MS, Hsu CH, Chang WT, Huang AM (2013) Novel genes that mediate nuclear respiratory factor 1-regualted neurite outgrowth in neuroblastoma IMR-32 cells. Gene 515:62-70.

Tripathy R et al. (2018) Mutations in MAST1 Cause Mega-Corpus-Callosum Syndrome with Cerebellar Hypoplasia and Cortical Malformations. Neuron 100:1354-+.

van Leyen K, Arai K, Jin G, Kenyon V, Gerstner B, Rosenberg PA, Holman TR, Lo EH (2008) Novel lipoxygenase inhibitors as neuroprotective reagents. J Neurosci Res 86:904-909.

Villa C et al. (2011) Role of hnRNP-A1 and miR-590-3p in Neuronal Death: Genetics and Expression Analysis in Patients with Alzheimer Disease and Frontotemporal Lobar Degeneration. Rejuv Res 14:275-281.

Wang H, Duan S, Du J, Li X, Xu Y, Zhang Z, Wang Y, Huang G, Feng G, He L (2006) Transmission disequilibrium test provides evidence of association between promoter polymorphisms in 22q11 gene DGCR14 and schizophrenia. J Neural Transm 113:1551-1561.

Wang SM, Yang WL (2009) Circulating hormone adrenomedullin and its binding protein protect neural cells from hypoxia-induced apoptosis. Bba-Gen Subjects 1790:361-367.

Wee CD, Havens MA, Jodelka FM, Hastings ML (2014) Targeting SR Proteins Improves SMN Expression in Spinal Muscular Atrophy Cells. Plos One 9.

Werneburg S, Fuchs HLS, Albers I, Burkhardt H, Gudi V, Skripuletz T, Stangel M, Gerardy-Schahn R, Hildebrandt H (2017) Polysialylation at Early Stages of Oligodendrocyte Differentiation Promotes Myelin Repair. J Neurosci 37:8131-8141.

Woo JS, Kim VN (2014) MeCP2 Caught Moonlighting as a Suppressor of MicroRNA Processing. Dev Cell 28:477-478.

Xie Y, Hou W, Song X, Yu Y, Huang J, Sun X, Kang R, Tang D (2016) Ferroptosis: process and function. Cell Death Differ 23:369-379.

Yao J, Ong SE, Bajjalieh S (2014) Huntingtin is associated with cytomatrix proteins at the presynaptic terminal. Mol Cell Neurosci 63:96-100.

Yigitkanli K, Zheng Y, Pekcec A, Lo EH, van Leyen K (2017) Increased 12/15-Lipoxygenase Leads to Widespread Brain Injury Following Global Cerebral Ischemia. Transl Stroke Res 8:194-202.

Yu J, Li X, Matei N, McBride D, Tang JP, Yan M, Zhang JH (2018) Ezetimibe, a NPC1L1 inhibitor, attenuates neuronal apoptosis through AMPK dependent autophagy activation after MCAO in rats. Exp Neurol 307:12-23.

Zhao JJ, Zhang P, He Z, Chen SX, Golden T, Li L, Li MZ, Wu N (2018) The stress response HPA-axis hormone, glucocorticoid, reduces cellular SKA complex gene expression. Psychiat Res 260:428-431.
