# Supplementary material for: Expression Signature of lncRNAs and mRNAs in Sevoflurane-Induced Mouse Brain Injury: Implication of Involvement of Wide Molecular Networks and Pathways
Source: Int J Mol Sci. 2021 Jan 30;22(3):1389. doi: 10.3390/ijms22031389 (PMC7869012; doi:10.3390/ijms22031389)
Supplement: Supplementary file 1 [file ijms-22-01389-s001.zip › ijms-1088385-supplementary/5. Supplementary Table S2 10.11.docx]

Supplementary Table 3. The bioinformatic analysis of predicted neurological diseases and functions of the sevoflurane-induced dysregulated mRNA profiles using Ingenuity Pathway Analysis software.

| **Neurological disease or**  **function annotation** | **P-value (Fisher’s exact test)** | **Sevoflurane-induced dysregulated mRNAs** |
| --- | --- | --- |
| Apoptosis of granule cells | 1.2E-05 | ADCYAP1, FAS, FRAT1, JUN, MECP2 |
| Epilepsy or neurodevelopmental disorder | 4.7E-05 | ADCYAP1, AHNAK, BRPF1, CCR1, DUSP5, EGR4,  GLI3, HAS3, HSPB3, JUN, MECP2, NR4A3, SCN11A |
| Epileptic seizure | 7E-05 | ADCYAP1, CCR1, DUSP5, EGR4, HAS3, HSPB3, JUN, NR4A3 |
| Epilepsy | 8E-05 | ADCYAP1, CCR1, DUSP5, EGR4, GLI3, HAS3, HSPB3, JUN, MECP2, NR4A3, SCN11A |
| Apoptosis of cerebellar granule cell | 0.00019 | FRAT1, JUN, MECP2 |
| Cell death of cerebellar cortex cells | 0.00024 | FAS, FRAT1, JUN, MECP2 |
| Apoptosis of brain cells | 0.00044 | ADCYAP1, ALOX15, FAS, FRAT1, JUN, MECP2 |
| Seizures | 0.00087 | ADCYAP1, CCR1, DUSP5, EGR4, HAS3, HSPB3, JUN, MECP2, NR4A3, SCN11A, ST8SIA4 |
| Seizure disorder | 0.00088 | ADCYAP1, CCR1, DUSP5, EGR4, GLI3, HAS3, HSPB3, JUN, MECP2, NR4A3, SCN11A, ST8SIA4 |
| Apoptosis of cortical neurons | 0.00248 | ALOX15, FAS, JUN, MECP2 |
| Degenerative ataxia | 0.00366 | FAS, IGFBP5, SCN11A |
| **Neurological disease or**  **function annotation** | **P-value (Fisher’s exact test)** | **Sevoflurane-induced dysregulated mRNAs** |
| Hereditary sensory and autonomic neuropathy type VII | 0.00541 | SCN11A |
| Zappella variant Rett syndrome | 0.00541 | MECP2 |
| Blepharophimosis, ptosis, and epicanthus inversus type II with Duane retraction syndrome | 0.00541 | FOXL2 |
| Kallmann syndrome type 17 | 0.00541 | SPRY4 |
| Susceptibility to X-linked autism type 3 | 0.00541 | MECP2 |
| Distal hereditary motor neuronopathy type IIC | 0.00541 | HSPB3 |
| Susceptibility to hypogonadotropic hypogonadism type 17 | 0.00541 | SPRY4 |
| Neurodegeneration of sympathetic neuron | 0.00541 | JUN |
| Hypogonadotropic hypogonadism 17 without anosmia | 0.00541 | SPRY4 |
| X-linked mental retardation syndromic 13 | 0.00541 | MECP2 |
| Neonatal severe encephalopathy | 0.00541 | MECP2 |
| Intellectual developmental disorder with dysmorphic facies and ptosis | 0.00541 | BRPF1 |
| Degeneration of cholinergic fibers | 0.00541 | JUN |
| **Neurological disease or**  **function annotation** | **P-value (Fisher’s exact test)** | **Sevoflurane-induced dysregulated mRNAs** |
| Familial episodic pain syndrome type 3 | 0.00541 | SCN11A |
| Lubs X-linked mental retardation syndrome | 0.00541 | MECP2 |
| Hypothalamic hamartoma | 0.00541 | GLI3 |
| Anosmia | 0.0107 | SLC7A11, SPRY4 |
| Apoptosis of somatotrophs | 0.0108 | FAS |
| Apoptosis of lactotropes | 0.0108 | FAS |

*Note: The full gene names of the sevoflurane-induced dysregulated mRNAs are detailed in Supplementary Table 6.*
