# Supplementary material for: Expression Signature of lncRNAs and mRNAs in Sevoflurane-Induced Mouse Brain Injury: Implication of Involvement of Wide Molecular Networks and Pathways
Source: Int J Mol Sci. 2021 Jan 30;22(3):1389. doi: 10.3390/ijms22031389 (PMC7869012; doi:10.3390/ijms22031389)
Supplement: Supplementary file 1 [file ijms-22-01389-s001.zip › ijms-1088385-supplementary/7. Supplementary Table S5.docx]

Supplementary Table 5. The bioinformatics analysis of co-expressed sevoflurane-dysregulated lncRNA and mRNAs involved in apoptosis and necrosis signaling networks.

| **mRNAs** | | | | **Highly correlated co-expressed lncRNAs** | |  |  |  |  |  |
| --- | --- | --- | --- | --- | --- | --- | --- | --- | --- | --- |
| DUSP5 | | | | | AK131720, AK167175 | | | | | |
| EGR4 | | | \| ENSMUST00000134436, NR_024257,NR_036459, AK131720, uc009alp.2, ENSMUST00000126693, AK045957, AK086925, AV471140, ENSMUST00000155363, AK036595, AK014666, AK167175, ENSMUST00000145890, ENSMUST00000137546, AK045892, uc007uqa.1, NR_045190 \| \| --- \| | | | | | | |  |
| ITK | | | \| NR_024257, AK131720, ENSMUST00000169975, AK045957, AV471140, AK143771, AK167175, AK081961, AK030101, ENSMUST00000137546, mouselincRNA0733-, NR_045190 \| \| --- \| | | | | | | |  |
| JUN | | | \| AK143771, AK167175, AK081961, AK030101, ENSMUST00000137546, mouselincRNA0733- \| \| --- \| | | | | | | |  |
| MECP2 | | | \| NR_024257, AK131720, AV471140, AK143771, AK167175, AK081961, AK030101, ENSMUST00000137546, mouselincRNA0733- \| \| --- \| | | | | | | |  |
| NR4A3 | | \| ENSMUST00000169975, AK143771, TCONS_00020794, ENSMUST00000137546, ENSMUST00000135739, ENSMUST00000172524, uc012fts.1, uc009mzl.1, TCONS_00000533 \| \| --- \| | | | | | | |  |  |
| PIANP | | \| 2_00012457, TCONS_00003993, uc012fts.1, uc009mzl.1,ENSMUST00000117770, AK084576, NR_045190 \| \| --- \| | | | | | | |  |  |
| PIK3R1 | | \| ENSMUST00000134436, NR_024257, NR_036459, AK131720, uc009alp.2, ENSMUST00000169975, AK045957, AV471140, AK143771, AK036595, AK167175, AK081961, ENSMUST00000145890, ENSMUST00000137546, ENSMUST00000147578, mouselincRNA0733- \| \| --- \| | | | | | |  |  |  |
| S1PR4 | | \| ENSMUST00000135739, ENSMUST00000172524, uc007guo.1, AK032934 \| \| --- \| | | | | | |  |  |  |
| **mRNAs** | **Highly correlated co-expressed lncRNAs** | | | | | |  |  |  |  |
| SLC40A1 | \| AK045957, AK143771, AK167175, AK081961, ENSMUST00000137546, ENSMUST00000141521, ENSMUST00000147578, mouselincRNA0733-, AK032934 \| \| --- \| | | | | | |  |  |  |  |
| SLC7A11 | \| ENSMUST00000141521, ENSMUST00000135739, uc007guo.1, ENSMUST00000117770, AK032934 \| \| --- \| | | | | | |  |  |  |  |
| SLPI | \| AK131720, uc009alp.2, AK045957, AV471140, AK036595, AK167175, AK081961 \| \| --- \| | | | | | |  |  |  |  |
| ST8SIA4 | \| ENSMUST00000118347, TCONS_00020794 \| \| --- \| | | | | | |  |  |  |  |
| Srsf5 | uc007guo.1 | | | | | |  |  |  |  |
| TNFRSF11B | ENSMUST00000141521 | | | | | |  |  |  |  |
| ADCYAP1 | \| ENSMUST00000153752, uc012fts.1, AK084576 \| \| --- \| | | | | | |  |  |  |  |
| DUSP5 | AK131720, AK167175 | | | | | |  |  |  |  |
| EGR4 | \| ENSMUST00000134436, NR_024257,NR_036459, AK131720, uc009alp.2, ENSMUST00000126693, AK045957, AK086925, AV471140, ENSMUST00000155363, AK036595, AK014666, AK167175, ENSMUST00000145890, ENSMUST00000137546, AK045892, uc007uqa.1, NR_045190 \| \| --- \| | | | | | |  |  |  |  |
| ITK | \| NR_024257, AK131720, ENSMUST00000169975, AK045957, AV471140, AK143771, AK167175, AK081961, AK030101, ENSMUST00000137546, mouselincRNA0733-, NR_045190 \| \| --- \| | | | | | |  |  |  |  |
| JUN | \| AK143771, AK167175, AK081961, AK030101, ENSMUST00000137546, mouselincRNA0733- \| \| --- \| | | | | | |  |  |  |  |
| **mRNAs** | **Highly correlated co-expressed lncRNAs** | | | | | |  |  |  |  |
| MECP2 | \| NR_024257, AK131720, AV471140, AK143771, AK167175, AK081961, AK030101, ENSMUST00000137546, mouselincRNA0733- \| \| --- \| | | | | | |  |  |  |  |
| NR4A3 | \| ENSMUST00000169975, AK143771, TCONS_00020794, ENSMUST00000137546, ENSMUST00000135739, ENSMUST00000172524, uc012fts.1, uc009mzl.1, TCONS_00000533 \| \| --- \| | | | | | |  |  |  |  |
| PIANP | \| 2_00012457, TCONS_00003993, uc012fts.1, uc009mzl.1,ENSMUST00000117770, AK084576, NR_045190 \| \| --- \| | | | | | |  |  |  |  |
| PIK3R1 | \| ENSMUST00000134436, NR_024257, NR_036459, AK131720, uc009alp.2, ENSMUST00000169975, AK045957, AV471140, AK143771, AK036595, AK167175, AK081961, ENSMUST00000145890, ENSMUST00000137546, ENSMUST00000147578, mouselincRNA0733- \| \| --- \| | | | | | |  |  |  |  |
| S1PR4 | \| ENSMUST00000135739, ENSMUST00000172524, uc007guo.1, AK032934 \| \| --- \| | | | | | |  |  |  |  |
| SLC40A1 | AK045957, AK143771, AK167175, AK081961, ENSMUST00000137546, ENSMUST00000141521, ENSMUST00000147578, mouselincRNA0733-, AK032934 | | | | | |  |  |  |  |

*Note: The full gene names are detailed in Supplementary Table 6.*
